# Risk Stratification and Management of Advanced Conduction Disturbances Following TAVI in Patients With Pre-Existing RBBB

**DOI:** 10.1016/j.shj.2022.100006

**Published:** 2022-03-17

**Authors:** Toshiaki Isogai, Iryna Dykun, Ankit Agrawal, Shashank Shekhar, Anas M. Saad, Beni Rai Verma, Omar M. Abdelfattah, Ankur Kalra, Amar Krishnaswamy, Grant W. Reed, Samir R. Kapadia, Rishi Puri

**Affiliations:** a Department of Cardiovascular Medicine, Heart, Vascular and Thoracic Institute, Cleveland Clinic, Cleveland, Ohio, USA; b Department of Cardiology and Vascular Medicine, West German Heart and Vascular Center, University of Duisburg-Essen, Essen, Germany

**Keywords:** Complete heart block, Implantation depth, Permanent pacemaker, Right bundle branch block, Transcatheter aortic valve implantation

## Abstract

**Background:**

Pre-existing right bundle branch block (RBBB) is a strong predictor of increased need for a permanent pacemaker (PPM) following transcatheter aortic valve implantation (TAVI). Yet, further risk stratification and management remain challenging in patients with pre-existing RBBB owing to limited data. Therefore, we sought to investigate the incidence, predictors, and management of advanced conduction disturbances after TAVI in patients with pre-existing RBBB.

**Methods:**

We retrospectively reviewed 261 consecutive patients with pre-existing RBBB (median age 81 years; 28.0% female; 95.0% received a balloon-expandable valve) without a pre-existing PPM who underwent TAVI at our institution in 2015-2019. Outcomes were high-degree atrioventricular block/complete heart block (HAVB/CHB) and PPM requirement.

**Results:**

Overall, the 30-day HAVB/CHB rate was 28.0%, of which 76.7% occurred during the TAVI procedure. The delayed HAVB/CHB rate was 8.3%. Implantation depth below aortic annulus (per 1-mm increase) was significantly associated with increased risk of procedural HAVB/CHB (adjusted odds ratio = 1.25, 95% confidence interval = 1.07-1.46), delayed HAVB/CHB (1.34 [1.01-1.79]), and 30-day PPM (1.32 [1.11-1.55]). Predilation was associated with delayed HAVB/CHB (4.02 [1.22-13.23]). The combination of no predilation and implantation depth of ≤2.0 mm had lower rates of procedural HAVB/CHB (11.2% vs. 26.7%-30.4%, *p* = 0.011), delayed HAVB/CHB (2.1% vs. 7.6%-28.1%, *p* < 0.001), and 30-day PPM (10.3% vs. 20.0%-43.5%, *p* < 0.001) than the other strategies of valve deployment. Complete HAVB/CHB recovery after PPM implantation was uncommon at 7.1%.

**Conclusions:**

In patients with pre-existing RBBB, the majority of HAVB/CHB events occurred during the TAVI procedure. Avoidance of predilation coupled with high valve deployment may result in relatively low rates of procedural and delayed HAVB/CHB, along with 30-day PPM rates.

## Introduction

With technological advances and established clinical evidence during the last decade, transcatheter aortic valve implantation (TAVI) is now the preferred intervention for the majority of patients with severe aortic stenosis.^1^ Yet, the occurrence of advanced conduction disturbances following TAVI remains a major issue, owing to anatomical proximity of the positioned oversized transcatheter heart valve (THV) prosthesis and the atrioventricular node/His-Purkinje system.^2^, ^3^, ^4^ Despite newer-generation THVs, average permanent pacemaker (PPM) rates remain around 10% or higher at 30 days after TAVI.^5^^,^^6^ Importantly, pre-existing right bundle branch block (RBBB) is the most consistent patient factor that predicts PPM risk.^7^ A recent meta-analysis revealed an increased 30-day PPM risk in patients with versus without pre-existing RBBB (38.1% vs. 11.4%; risk ratio = 3.56).^8^ Furthermore, recent studies using ambulatory monitoring devices revealed pre-existing RBBB as a predictor of delayed conduction disorders after TAVI.^9^^,^^10^

The prevalence of RBBB is up to 3% in the general population^11^^,^^12^ but increases with age.^13^ Accordingly, pre-existing RBBB is more common (5%-20%) among patients undergoing aortic valve replacement.^8^^,^^14^ Despite the high PPM risk after TAVI in patients with pre-existing RBBB, appropriate management has yet to be established owing to scarce data on detailed clinical course after TAVI.^2^^,^^3^ Moreover, while numerous studies have demonstrated pre-existing RBBB *per se* as an overall strong predictor of PPM requirement after TAVI,^7^^,^^8^ few data exist on additional predictors to further optimize risk stratification in this high PPM risk subpopulation. Therefore, the present study sought to evaluate the incidence, timing, predictors, and management of advanced conduction disturbances after TAVI in patients with pre-existing RBBB. A key aim of this analysis was to further identify secondary risk factors that consolidate advanced conduction disturbances and PPM risk in TAVI recipients with pre-existing RBBB.

## Materials and Methods

### Study Design and Data Collection

We undertook a retrospective analysis of consecutive patients aged ≥18 years with pre-existing RBBB (QRS duration ≥120 msec) who underwent TAVI at Cleveland Clinic between January 2015 and December 2019. Patients with pre-existing cardiac implantable electronic devices were excluded. Data on patient characteristics, electrocardiogram (ECG), imaging data, procedural characteristics, and in-hospital/postdischarge adverse events were extracted from our prospective institutional registries or were manually collected from electronic medical records. Adverse events were based on the Valve Academic Research Consortium-2 criteria.^15^ The present study was approved by the Institutional Review Board of Cleveland Clinic with a waiver of informed consent owing to the retrospective nature of the study. The present research has adhered to the relevant ethical guidelines.

### Procedural Strategy and Postprocedure Management

In our institution, a high valve deployment technique has been introduced to decrease the post-TAVI PPM risk since April 2017.^16^ Briefly, the right anterior oblique (RAO) caudal view that removes the parallax from the inflow of the valve stent frame was used to confirm that the radiographic lucent line of the Edwards Sapien 3 THV or inflow of the Medtronic Evolut THV was positioned at or just below the base of the noncoronary cusp (NCC) before deployment. The THV was deployed while the position was maintained during the valve expansion. This technique allowed us to deploy the valve at a higher position than conventional deployment technique using a 10:0 to 8.5:1.5 ratio of the valve frame in the aorta:left ventricular outflow tract (LVOT).

The temporary pacemaker (TPM) was removed at the end of the TAVI procedure irrespective of pre-existing conduction disturbances unless high-degree atrioventricular block (HAVB) or complete heart block (CHB) occurred periprocedurally or maintaining a TPM was deemed necessary by the operating physicians. When HAVB/CHB or bradyarrhythmia was observed during continuous telemetry monitoring after TAVI, TPM re-insertion was considered, and our electrophysiology team was consulted for PPM requirement. A Zio Patch (iRhythm San Francisco, CA) could be initiated upon hospital discharge for ambulatory ECG monitoring at the discretion of operating physicians or as part of our prior prospective study.^17^ In our institution, outpatient follow-up (typically with nurse practitioners or physician assistants) was routinely arranged within 3-7 days after discharge for all patients who underwent TAVI.

### ECG Assessment

Twelve-lead (or 6-lead) ECGs were routinely undertaken at least at 3 different time points (at baseline before TAVI, immediately after TAVI, and day 1 after TAVI). All ECG and telemetry data were evaluated according to the standard definitions and guidelines by the American Heart Association, American College of Cardiology Foundation, and Heart Rhythm Society recommendations.^18^^,^^19^ HAVB/CHB was divided into procedural HAVB/CHB and delayed HAVB/CHB according to a recent expert panel document^3^: the former was defined as any HAVB/CHB episode occurring during the TAVI procedure, while the latter was defined as any HAVB/CHB episode occurring after the patient had left the procedure room and within 30 days after the procedure. Procedural HAVB/CHB was further subdivided into procedural persistent HAVB/CHB (defined as HAVB/CHB that persisted until the end of the TAVI procedure) and procedural transient HAVB/CHB (defined as HAVB/CHB that recovered by the end of the procedure).

### Imaging Assessment

Data on aortic annulus were measured using ECG-gated computed tomography (CT) images with contrast before TAVI (or cardiac magnetic resonance imaging if contrast CT images were unavailable owing to poor renal function). The calcium score of aortic valve leaflets was quantified using ECG-gated contrast CT images before TAVI. A prespecified threshold was set to account for the hyperdensity of the applied contrast medium according to a previous report.^20^ The presence or absence of LVOT calcification was also examined using available contrast-enhanced or noncontrast CT images before TAVI. These measurements were performed using Aquarius iNtuition (TeraRecon Inc, Foster City, CA). The eccentricity index and oversizing were calculated based upon the methods of a prior study.^21^ Implantation depth of the THV relative to the base of the NCC was defined as the distance between the bottoms of the NCC and the valve stent frame in the final RAO caudal aortic root angiogram and was measured using SyngoDynamics imaging software (Siemens Healthcare, Malvern, PA).

### Outcome Measures

Outcomes of interest were the occurrence of procedural and delayed HAVB/CHB, TPM reinsertion, and the requirement of the PPM or implantable cardioverter defibrillator (ICD) within 30 days after TAVI. In-hospital adverse events and postdischarge outcomes were also assessed. Major adverse cardiovascular events (MACE) were defined as all-cause death, stroke/transient ischemic attack, and hospitalization for heart failure. In PPM/ICD recipients, the right ventricular (RV) pacing rate was assessed at 1-3 months after PPM/ICD implantation. Complete HAVB/CHB recovery was defined as an RV pacing rate of <1%, and PPM dependency was defined as an RV pacing rate of >40% according to a recent study.^22^

### Statistical Analysis

Categorical variables were presented as numbers and percentages and were compared using the chi-square test or Fisher exact test. Continuous variables were presented as median and interquartile range (IQR) or mean ± standard deviation and were compared using the Mann-Whitney *U* test or Student *t*-test as appropriate. Multivariable logistic regression analyses were conducted to identify independent predictors of procedural HAVB/CHB, delayed HAVB/CHB, and 30-day PPM/ICD requirement. In each multivariable analysis, variables with clinical interest found to be *p* < 0.05 in univariable analyses were entered as covariates. Missing data were handled with multiple imputation, while complete case analyses were also conducted. We also performed a sensitivity analysis for those predictors using only patients who underwent TAVI with a balloon-expandable valve. The C-statistic, the area under the receiver operating characteristic curve, was calculated to determine the predictive ability for HAVB/CHB and 30-day PPM/ICD requirement. Kaplan–Meier curves were used to compare death and MACE between patients with or without the 30-day PPM/ICD with the use of the log-rank test. A 2-sided *p*-value of <0.05 was considered significant in all hypothesis tests. All statistical analyses were conducted using IBM SPSS Statistics, version 27 (IBM Corp, Armonk, NY).

## Results

### Study Patients

Among 2296 patients who underwent TAVI between January 2015 and December 2019, 261 patients (11.4%) with pre-existing RBBB without pre-existing cardiac implantable electronic devices were identified, comprising the present study cohort (Supplemental Figure 1). The median age was 81.0 years; 28.0% were women; the median Society of Thoracic Surgeons (STS) risk score was 5.14%. Overall, 13.4% of the patients had atrial fibrillation (AF) at baseline before TAVI, 33.3% had first degree atrioventricular block, 26.8% had left anterior fascicular block, and 1.1% had left anterior fascicular block. As a result, 28.0% had bifascicular block, and 9.2% had trifascicular block (Table 1). A transfemoral approach was used in 93.5% of the patients (17 nontransfemoral: 7 subclavian, 7 apical, and 3 direct aortic). Balloon-expandable valves were used in 95.0% of the patients (237 Sapien 3; 11 Sapien XT: Edwards Lifesciences, Irvine, California), and self-expanding THVs were used in 5.0% of the patients (1 Evolut PRO; 9 Evolut R; 3 CoreValve: Medtronic, Minneapolis, Minnesota).

**Table 1:** Baseline and procedural characteristics of patients who did and did not develop procedural HAVB/CHB

	All	Procedural HAVB/CHB		*p* value
	(N = 261)	No (n = 205)	Yes (n = 56)	
Age, y	81.0 (76.0-86.0)	81.0 (76.0-85.0)	84 (77.5-88.0)	0.086
Female	73 (28.0)	52 (25.4)	21 (37.5)	0.092
Caucasian	251 (96.2)	197 (96.1)	54 (96.4)	1.00
Body mass index, kg/m^2^	29.1 (25.4-33.3)	29.4 (25.4-33.6)	27.5 (25.4-32.7)	0.36
STS risk score, %	5.14 (3.50-8.06)	4.62 (3.32-7.97)	5.85 (4.35-10.84)	0.006
Prior CABG	83 (31.8)	69 (33.7)	14 (25.0)	0.26
Prior myocardial infarction	63 (24.1)	47 (22.9)	16 (28.6)	0.38
ESRD on dialysis	12 (4.6)	7 (3.4)	5 (8.9)	0.14
Chronic lung disease	132 (50.6)	101 (49.3)	31 (55.4)	0.45
History of syncope	18 (6.9)	13 (6.3)	5 (8.9)	0.55
History of atrial fibrillation/flutter	101 (38.7)	74 (36.1)	27 (48.2)	0.12
NYHA functional class III or IV	210 (80.5)	163 (79.5)	47 (83.9)	0.57
LVEF, %	59 (54-63)	59 (55-64)	56 (50-63)	0.074
Aortic valve area, cm^2^	0.71 (0.58-0.84) [N = 245]	0.72 (0.61-0.85) [n = 189]	0.64 (0.52-0.79) [n = 56]	0.007
Aortic valve mean gradient, mmHg	41 (34-52)	41 (34-51)	46 (35.5-56)	0.30
Aortic valve peak gradient, mmHg	70 (58-86)	70 (58-85)	71 (58-89)	0.71
Bicuspid aortic valve	14 (5.4)	10 (4.9)	4 (7.1)	0.51
Failed bioprosthetic valve	18 (6.9)	17 (8.3)	1 (1.8)	0.13
Moderate or severe AR	48 (18.4)	39 (19.0)	9 (16.1)	0.70
Data on aortic annulus∗	[N = 253]	[n = 197]	[n = 56]	
Maximum annular diameter, mm	28 (26-30)	28 (26-30)	28 (26-30.5)	0.85
Minimum annular diameter, mm	23 (21-25)	23 (21-24.9)	22.5 (20.5-25)	0.62
Eccentricity index	0.18 (0.14-0.23)	0.18 (0.14-0.23)	0.19 (0.15-0.25)	0.28
Annular area, mm^2^	493 (416-560)	500 (420-560)	476 (411-560)	0.67
Calcium score of aortic valve leaflets, HU†	2118 (1310-3261) [N = 197]	2168 (1321-3240) [n = 149]	2051 (1263-3345) [n = 48]	0.80
LVOT calcification‡	135/241 (56.0)	98/186 (52.7)	37/55 (67.3)	0.064
Pre-TAVI baseline ECG findings				
Rhythm				0.87
Sinus rhythm	218 (83.5)	173 (84.4)	45 (80.4)	
Atrial fibrillation	35 (13.4)	26 (12.7)	9 (16.1)	
Atrial flutter	5 (1.9)	4 (2.0)	1 (1.8)	
Junctional rhythm	3 (1.1)	2 (1.0)	1 (1.8)	
PR interval, ms	190 (170-220) [N = 220]	190 (168-219) [n = 175]	190 (172-226) [n = 45]	0.56
QRS duration, ms	146 (136-156)	146 (136-156)	145 (137-153)	0.72
First degree AVB	87 (33.3)	68 (33.2)	19 (33.9)	1.00
QRS duration ≥150 ms	109 (41.8)	87 (42.4)	22 (39.3)	0.76
Left anterior fascicular block	70 (26.8)	57 (27.8)	13 (23.2)	0.61
Left posterior fascicular block	3 (1.1)	3 (1.5)	0 (0.0)	1.00
Bifascicular block	73 (28.0)	60 (29.3)	13 (23.2)	0.41
Trifascicular block	24 (9.2)	19 (9.3)	5 (8.9)	1.00
Procedural details				
Nonelective procedure	12 (4.6)	10 (4.9)	2 (3.6)	1.00
Nonfemoral approach	17 (6.5)	12 (5.9)	5 (8.9)	0.37
Anesthesia type				0.24
Conscious sedation	215 (82.4)	172 (83.9)	43 (76.8)	
General anesthesia	46 (17.6)	33 (16.1)	13 (23.2)	
Valve type				0.49
Balloon-expandable	248 (95.0)	196 (95.6)	52 (92.9)	
Self-expanding	13 (5.0)	9 (4.4)	4 (7.1)	
Valve size				0.38
≤23 mm	73 (28.0)	55 (26.8)	18 (32.1)	
26 mm	100 (38.3)	83 (40.5)	17 (30.4)	
≥29 mm	88 (33.7)	67 (32.7)	21 (37.5)	
Predilation	62 (23.8)	44 (21.5)	18 (32.1)	0.11
Postdilation	118 (45.2)	91 (44.4)	27 (48.2)	0.65
Oversizing, %	5.0 (0.8-8.6) [N = 253]	4.8 (0.9-8.6) [n = 197]	5.7 (0.5-9.7) [n = 56]	0.40
Implantation depth relative to the NCC, mm§	2.3 (1.0-4.0) [N = 259]	2.0 (0.9-3.6) [n = 204]	3.1 (1.6-5.0) [n = 55]	<0.001

*Notes*. Values are n (%), n/total n (%), or median (interquartile range).

AR = aortic regurgitation, AV = aortic valve, AVB = atrioventricular block, CABG = coronary artery bypass grafting, CHB = complete heart block, CT = computed tomography, ECG = electrocardiogram, ESRD = end-stage renal disease, HAVB = high-degree atrioventricular block, HU = Hounsfield unit, LVEF = left ventricular ejection fraction, LVOT = left ventricular outflow tract, NCC = noncoronary cusp, NYHA = New York Heart Association, STS = Society of Thoracic Surgeons, TAVI = transcatheter aortic valve implantation.

∗ Data on aortic annulus were unavailable in 8 patients because neither contrast CT images nor cardiac magnetic resonance images were performed.

† The calcium score of aortic valve leaflets was unavailable in 64 patients owing to a lack of contrast CT images before TAVI or a prior bioprosthetic valve.

‡ Data on LVOT calcification were unavailable in 20 patients owing to a lack of appropriate CT images before TAVI or a prior bioprosthetic valve.

§ Implantation depth was unavailable in 2 patients owing to a lack of an appropriate fluoroscopy image.

**Figure 1:**
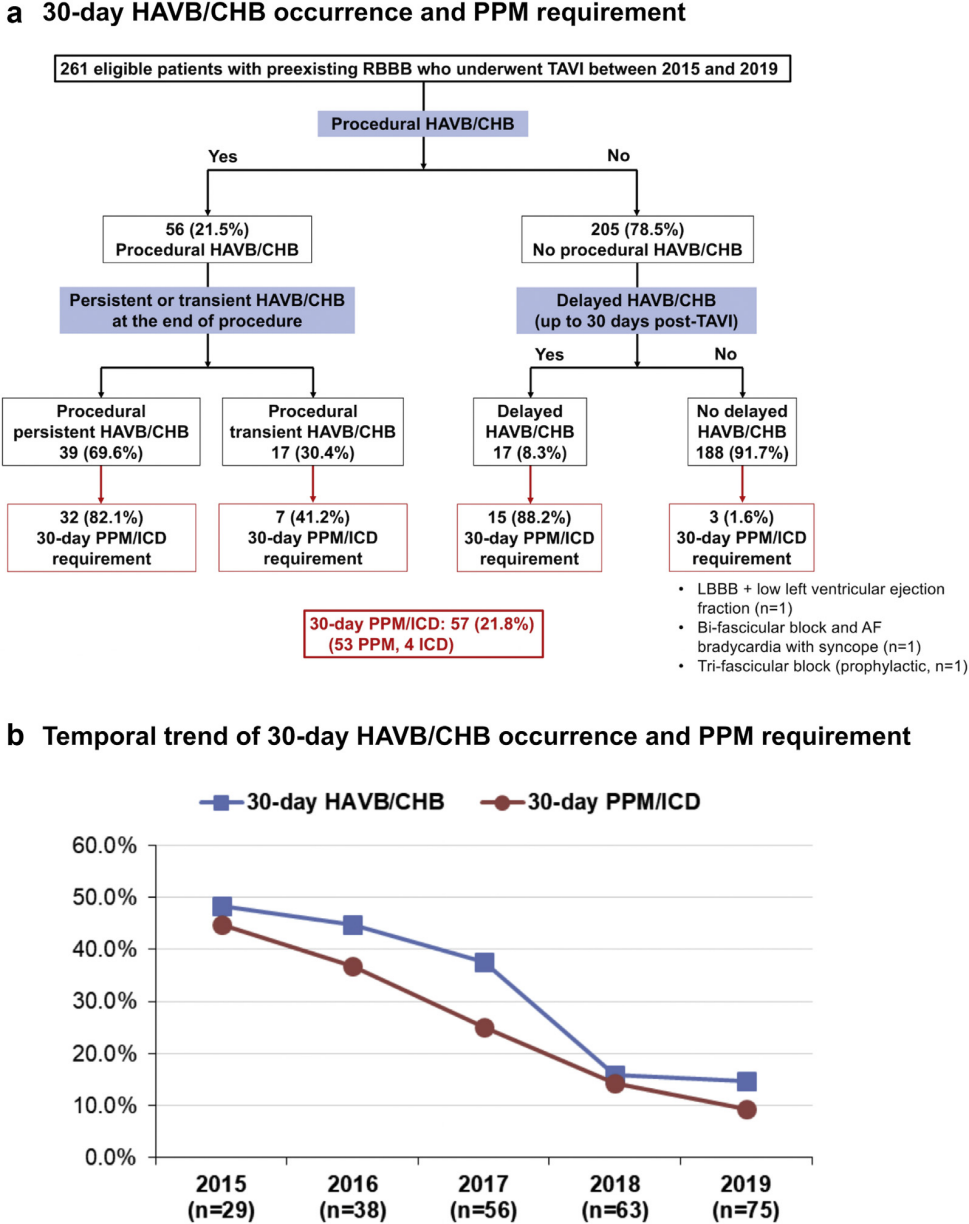
**Incidence and trend of 30-day HAVB/CHB and PPM requirement after TAVI in patients with pre-existing RBBB.** (a) The incidence of 30-day HAVB/CHB and PPM requirement after TAVI in patients with pre-existing RBBB. (b) The temporal trend of 30-day HAVB/CHB and PPM requirement after TAVI in patients with pre-existing RBBB between 2015 and 2019. Abbreviations: AF, atrial fibrillation; CHB, complete heart block; HAVB, high-degree atrioventricular block; ICD, implantable cardioverter defibrillator; LBBB, left bundle branch block; PPM, permanent pacemaker; RBBB, right bundle branch block; TAVI, transcatheter aortic valve implantation.

Of the 261 eligible patients, 56 (21.5%) patients developed procedural HAVB/CHB (39 patients developed procedural persistent, and 17 patients developed procedural transient); of the remaining 205 patients, 17 (8.3%) patients developed delayed HAVB/CHB (Figure 1a). Overall, 73 (28.0%) patients developed HAVB/CHB (70 CHBs and 3 HAVBs). The 30-day PPM/ICD rate was 21.8% (57/261) in patients with pre-existing RBBB, which was significantly higher than that in patients without pre-existing RBBB (6.4% [108/1692], *p* < 0.001), driven by the higher in-hospital PPM/ICD rate (20.7% vs. 5.3%, *p* < 0.001), with a similar postdischarge PPM/ICD rate (1.1% vs. 1.1%, *p* = 0.75). The incidence of 30-day HAVB/CHB and PPM requirement both declined over the 5 years from 2015 to 2019 in patients with pre-existing RBBB, to coincide in 2017 with the regular adoption of the NCC-guided valve implant from the RAO caudal view with subsequent high valve deployment technique (30-day HAVB/CHB, from 48.3% to 14.7%; 30-day PPM/ICD, from 44.8% to 9.3%; Figure 1b).^16^ The indications for the 30-day PPM/ICD were HAVB/CHB (n = 50), HAVB/CHB + low left ventricular ejection fraction (n = 4), left bundle branch block + low left ventricular ejection fraction (n = 1), bifascicular block and AF bradycardia with syncope (n = 1), and trifascicular block (prophylactic, n = 1). The types of implanted device were dual-chamber PPM (n = 49), single-chamber (RV lead) PPM (n = 2), leadless PPM (n = 1), and cardiac resynchronization therapy with a pacemaker (CRT-P, n = 1) or defibrillator (CRT-D, n = 4). A total of 27 patients were discharged with Zio Patch, which detected delayed HAVB/CHB in 2 patients. One patient was an 82-year-old man who underwent transfemoral TAVI with Sapien 3 (29 mm) with an implantation depth of 2.7 mm and developed symptomatic persistent CHB on day 4. The other patient was a 77-year-old man who underwent transfemoral TAVI with Sapien 3 (26 mm) with an implantation depth of 2.6 mm and developed symptomatic 5.5 sec pause owing to advanced atrioventricular block with permanent AF on day 13. Both HAVB/CHB events resulted in dual-chamber PPM implantation.

### Timing of 30-Day HAVB/CHB Occurrence and TPM Reinsertion

**Figure 2:**
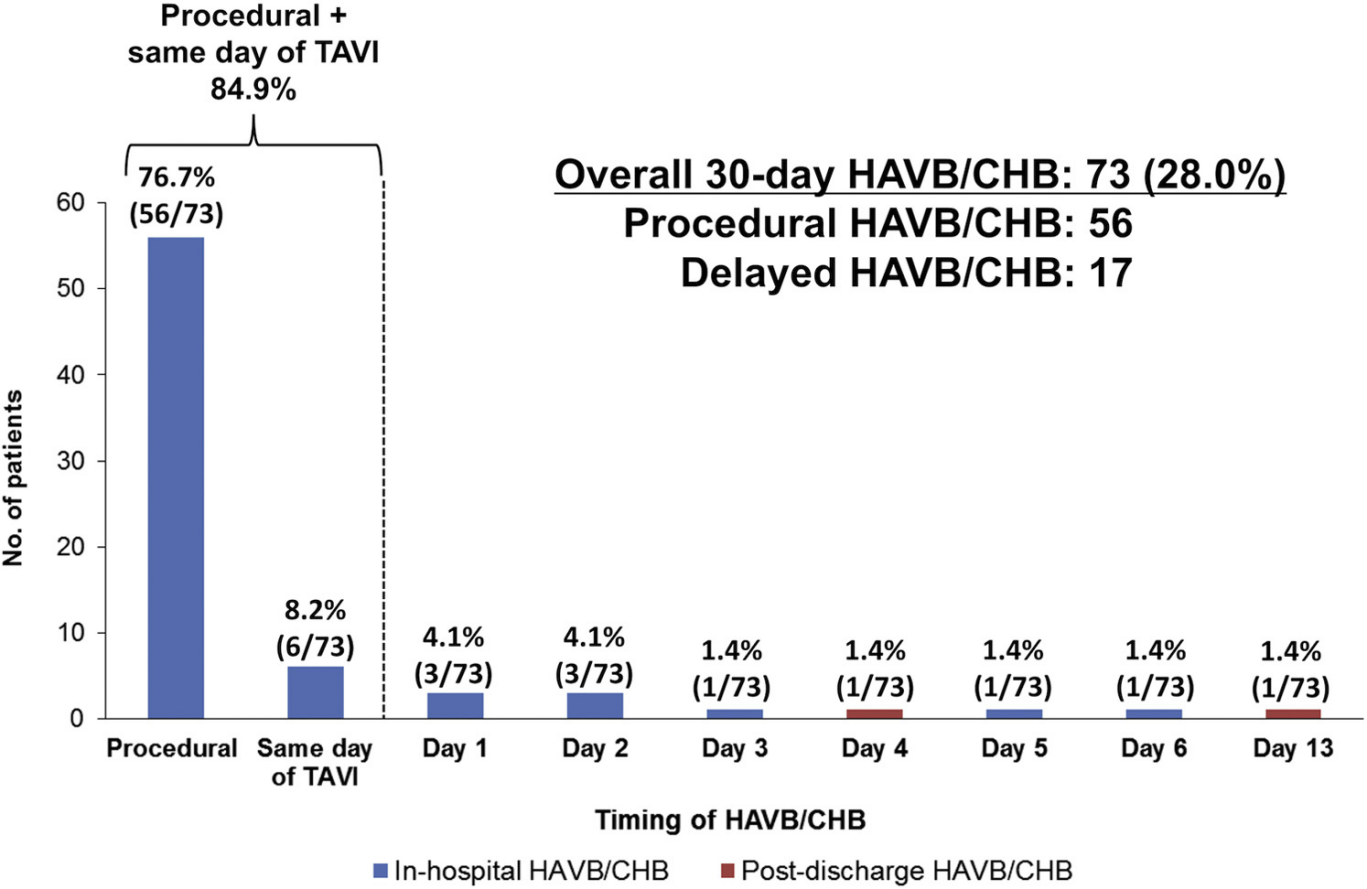
**Timing of 30-day HAVB/CHB occurrence in patients with pre-existing RBBB undergoing TAVI.** Abbreviations: CHB, complete heart block; HAVB, high-degree atrioventricular block; RBBB, right bundle branch block; TAVI, transcatheter aortic valve implantation.

The timing of HAVB/CHB occurrence ranged from procedural period to post-TAVI day 13 (Figure 2); the most frequent timing was the procedural period (76.7%), followed by the same day of TAVI (8.2%); 94.5% occurred within 3 days after TAVI. Two HAVB/CHB events occurred after discharge (day 4 and day 13).

In 205 patients without procedural HAVB/CHB, the TPM was removed at the end of the TAVI procedure in 199 (97.0%) patients, of whom 9 (4.5%) required TPM reinsertion. Details on these 9 patients are summarized in Supplemental Table 1. The reasons for TPM reinsertion in all patients were delayed HAVB/CHB events, including 8 CHBs and 1 Mobitz type II 2:1 atrioventricular block. When delayed HAVB/CHB was detected, 3 patients were symptomatic, and 6 patients were asymptomatic. No patient experienced cardiac arrest.

### Characteristics of Patients With HAVB/CHB and 30-Day PPM Requirement

Patients with procedural HAVB/CHB, as compared to those without, had a higher STS risk score, a smaller aortic valve area, and a greater implantation depth relative to the base of the NCC (Table 1). In a subgroup comparison between procedural persistent vs. procedural transient HAVB/CHB, although the age and the prevalence of the bicuspid aortic valve and first degree atrioventricular block appeared numerically different, the differences did not reach statistical significance (Supplemental Table 2). The 30-day PPM/ICD rate was significantly higher after procedural persistent HAVB/CHB than that after procedural transient HAVB/CHB (82.1% vs. 41.2%; *p* < 0.001).

Patients with delayed HAVB/CHB, as compared to those without, received general anesthesia and predilation more frequently and had a greater implantation depth (Table 2). Patients with 30-day PPM/ICD requirement, as compared to those without, had a greater prevalence of LVOT calcification and received a self-expanding valve and predilation more frequently, along with a greater implantation depth (Supplemental Table 3).

**Table 2:** Baseline and procedural characteristics of patients without procedural HAVB/CHB who did and did not develop delayed HAVB/CHB

	Delayed HAVB/CHB		*p* value
	No (n = 188)	Yes (n = 17)	
Age, y	81 (75-86)	81 (78-84)	0.89
Female	49 (26.1)	3 (17.6)	0.57
Caucasian	180 (95.7)	17 (100.0)	1.00
Body mass index, kg/m^2^	29.4 (25.3-33.6)	29.7 (27.5-33.3)	0.39
STS risk score, %	4.75 (3.32-8.04)	4.35 (3.32-5.80)	0.30
Prior CABG	63 (33.5)	6 (35.3)	1.00
Prior myocardial infarction	44 (23.4)	3 (17.6)	0.77
ESRD on dialysis	6 (3.2)	1 (5.9)	0.46
Chronic lung disease	93 (49.5)	8 (47.1)	1.00
History of syncope	10 (5.3)	3 (17.6)	0.081
History of atrial fibrillation/flutter	66 (35.1)	8 (47.1)	0.43
NYHA functional class III or IV	150 (79.8)	13 (76.5)	0.76
LVEF, %	59 (55-64)	57 (54-61)	0.66
Aortic valve area, cm^2^	0.72 (0.61-0.85) [n = 172]	0.75 (0.61-0.84) [n = 17]	0.87
Aortic valve mean gradient, mmHg	41 (33.5-51)	40 (34-46)	0.71
Aortic valve peak gradient, mmHg	70 (58-85)	72 (54-85)	0.99
Bicuspid aortic valve	9 (4.8)	1 (5.9)	0.59
Failed bioprosthetic valve	17 (9)	0 (0.0)	0.37
Moderate or severe AR	36 (19.1)	3 (17.6)	1.00
Data on aortic annulus	[n = 180]	[n = 17]	
Maximum annular diameter, mm	28 (26-30)	29 (27.6-30)	0.43
Minimum annular diameter, mm	23 (21-24)	23.8 (22-24)	0.85
Eccentricity index	0.18 (0.14-0.23)	0.21 (0.17-0.25)	0.32
Annular area, mm^2^	496.5 (410-570)	500 (460-540)	0.85
Calcium score of aortic valve leaflets, HU	2193 (1313-3340) [n = 134]	2096 (1331.5-3000) [n = 15]	0.77
LVOT calcification	89/169 (52.7)	9/17 (52.9)	1.00
Pre-TAVI baseline ECG findings			
Rhythm			0.29
Sinus rhythm	161 (85.6)	12 (70.6)	
Atrial fibrillation	22 (11.7)	4 (23.5)	
Atrial flutter	3 (1.6)	1 (5.9)	
Junctional rhythm	2 (1.1)	0 (0.0)	
PR interval, ms	188 (167-219) [n = 164]	202 (194-226) [n = 11]	0.16
QRS duration, ms	146 (136-156)	144 (128-156)	0.41
First degree AVB	62 (33.0)	6 (35.3)	1.00
QRS duration ≥150 ms	79 (42.0)	8 (47.1)	0.80
Left anterior fascicular block	52 (27.7)	5 (29.4)	1.00
Left posterior fascicular block	3 (1.6)	0 (0.0)	1.00
Bifascicular block	55 (29.3)	5 (29.4)	1.00
Trifascicular block	17 (9.0)	2 (11.8)	0.66
Procedural details			
Nonelective procedure	8 (4.3)	2 (11.8)	0.20
Nonfemoral approach	11 (5.9)	1 (5.9)	1.00
Anesthesia type			0.036
Conscious sedation	161 (85.6)	11 (64.7)	
General anesthesia	27 (14.4)	6 (35.3)	
Valve type			0.55
Balloon-expandable	180 (95.7)	16 (94.1)	
Self-expanding	8 (4.3)	1 (5.9)	
Valve size			0.10
≤23 mm	54 (28.7)	1 (5.9)	
26 mm	73 (38.8)	10 (58.8)	
≥29 mm	61 (32.4)	6 (35.3)	
Predilation	34 (18.1)	10 (58.8)	<0.001
Postdilation	86 (45.7)	5 (29.4)	0.21
Oversizing, %	4.6 (0.8-7.9) [n = 180]	7.4 (3.0-9.0) [n = 17]	0.12
Implantation depth relative to the NCC, mm	1.9 (0.8-3.5) [n = 187]	4.1 (2.6-5.8) [n = 17]	<0.001
ECG changes: end of TAVI - pre-TAVI			
ΔPR interval, ms	16 (2-31) [n = 155]	10 (−2 to 52) [n = 10]	0.92
ΔPR interval ≥20 ms	62 (40.0) [n = 155]	4 (40.0) [n = 10]	1.00
ΔQRS duration, ms	0 (−4 to 6)	−2 (−6 to 4)	0.80
ΔQRS duration ≥20 ms	2 (1.1)	1 (5.9)	0.23

*Notes*. Values are n (%), n/total n (%), or median (interquartile range).

AR = aortic regurgitation, AVB = atrioventricular block, CABG = coronary artery bypass grafting, CHB = complete heart block, ECG = electrocardiogram, ESRD = end-stage renal disease, HAVB = high-degree atrioventricular block, HU = Hounsfield unit, LVEF = left ventricular ejection fraction, LVOT = left ventricular outflow tract, NCC = noncoronary cusp, NYHA = New York Heart Association, STS = Society of Thoracic Surgeons, TAVI = transcatheter aortic valve implantation.

### In-Hospital Adverse Events

No patient died during the index hospitalization. A second valve deployment was needed in 5 patients for significant paravalvular leak (none of the second valve deployments were due to valve migration or embolization) after the first valve deployment, all of whom developed procedural HAVB/CHB. Overall, the median length of hospital stay was 3 (IQR = 2-5) days. The length of hospital stay was significantly longer in patients with than without HAVB/CHB. There were no significant differences in other in-hospital adverse events regardless of the occurrence of HAVB/CHB (Table 3).

**Table 3:** In-hospital adverse events

	All	Procedural HAVB/CHB			Delayed HAVB/CHB		
	(N = 261)	No (n = 205)	Yes (n = 56)	*p* value	No (n = 188)	Yes (n = 17)	*p* value
Death	0 (0.0)	0 (0.0)	0 (0.0)	(-)	0 (0.0)	0 (0.0)	(-)
Major vascular complication	1 (0.4)	0 (0.0)	1 (1.8)	0.21	0 (0.0)	0 (0.0)	(-)
Conversion to open surgery	0 (0.0)	0 (0.0)	0 (0.0)	(-)	0 (0.0)	0 (0.0)	(-)
Coronary obstruction	1 (0.4)	1 (0.5)	0 (0.0)	1.00	1 (0.5)	0 (0.0)	1.00
Second valve deployment	5 (1.9)	0 (0.0)	5 (8.9)	<0.001	0 (0.0)	0 (0.0)	(-)
Valve migration or embolization	0 (0.0)	0 (0.0)	0 (0.0)	(-)	0 (0.0)	0 (0.0)	(-)
New-onset atrial fibrillation	8 (3.1)	6 (2.9)	2 (3.6)	0.68	4 (2.1)	2 (11.8)	0.080
Paravalvular leak ≥2+	3 (1.1)	2 (1.0)	1 (1.8)	0.52	2 (1.1)	0 (0.0)	1.00
Stroke/transient ischemic attack	5 (1.9)	3 (1.5)	2 (3.6)	0.29	2 (1.1)	1 (5.9)	0.23
Overall length of stay, d	3 (2-5)	2 (1-4)	4 (3-8)	<0.001	2 (1-3)	4 (3-6)	<0.001
Post-TAVI length of stay, d	2 (2-4)	2 (1-3)	4 (3-7.5)	<0.001	2 (1-3)	3 (2-6)	<0.001

*Notes*. Values are n (%) or median (interquartile range).

CHB = complete heart block, HAVB = high-degree atrioventricular block, TAVI = transcatheter aortic valve implantation.

### Predictors for HAVB/CHB Occurrence and PPM Requirement

In multivariable analyses, greater implantation depth was independently associated with a higher risk of procedural HAVB/CHB, delayed HAVB/CHB, and 30-day PPM (Table 4 and Supplemental Table 4). The STS risk score and aortic valve area were also associated with a higher risk of procedural HAVB/CHB, while predilation was associated with a higher risk of delayed HAVB/CHB. These results with multiple imputations were consistent with those with complete case analyses (Supplemental Table 5). Moreover, those results were also consistent in the sensitivity analysis using only 248 patients who underwent TAVI with a balloon-expandable valve (Supplemental Figure 2 and Supplemental Tables 6 and 7).

**Table 4:** Predictors of HAVB/CHB and 30-d PPM/ICD requirement after TAVI in patients with pre-existing RBBB

	Univariable analyses			Multivariable analyses		
	OR	95% CI	*p* value	OR	95% CI	*p* value
Procedural HAVB/CHB (n = 261)						
STS risk score, per 1% increase	1.09	1.02-1.16	0.008	1.08	1.01-1.15	0.030
Aortic valve area, per 0.1-cm^2^ decrease	1.27	1.07-1.52	0.008	1.23	1.03-1.48	0.024
Implantation depth relative to the NCC, per 1-mm increase	1.27	1.09-1.48	0.002	1.25	1.07-1.46	0.004
Delayed HAVB/CHB (n = 205)∗						
General anesthesia (vs. conscious sedation)	3.25	1.11-9.53	0.032	1.03	0.29-3.68	0.96
Predilation	6.47	2.30-18.21	<0.001	4.02	1.22-13.23	0.022
Implantation depth relative to the NCC, per 1-mm increase	1.53	1.18-1.99	0.001	1.34	1.01-1.79	0.044
30-d PPM/ICD requirement (n = 261)						
LVOT calcification	2.37	1.24-4.53	0.009	1.77	0.89-3.52	0.11
Self-expanding valve (vs. balloon-expandable valve)	3.31	1.07-10.28	0.038	1.72	0.48-6.19	0.41
Predilation	3.18	1.69-5.98	<0.001	1.94	0.96-3.90	0.064
Implantation depth relative to the NCC, per 1-mm increase	1.42	1.21-1.66	<0.001	1.32	1.11-1.55	0.001

*Notes*. Predictors were examined in multivariable logistic regression models including variables with a *p* value at <0.05 in univariable models (Supplemental Table 3). In multivariable models, missing data for aortic valve area, implantation depth, and LVOT calcification were handled with multiple imputation.

CHB = complete heart block, CI = confidence interval, HAVB = high-degree atrioventricular block, ICD = implantable cardioverter defibrillator, LVOT = left ventricular outflow tract, NCC = noncoronary cusp, OR = odds ratio, PPM = permanent pacemaker, RBBB = right bundle branch block, TAVI = transcatheter aortic valve implantation.

∗ Includes patients who did not develop procedural HAVB/CHB.

### Predictive Values of Implantation Depth and Predilation for HAVB/CHB and PPM Requirement

**Figure 3:**
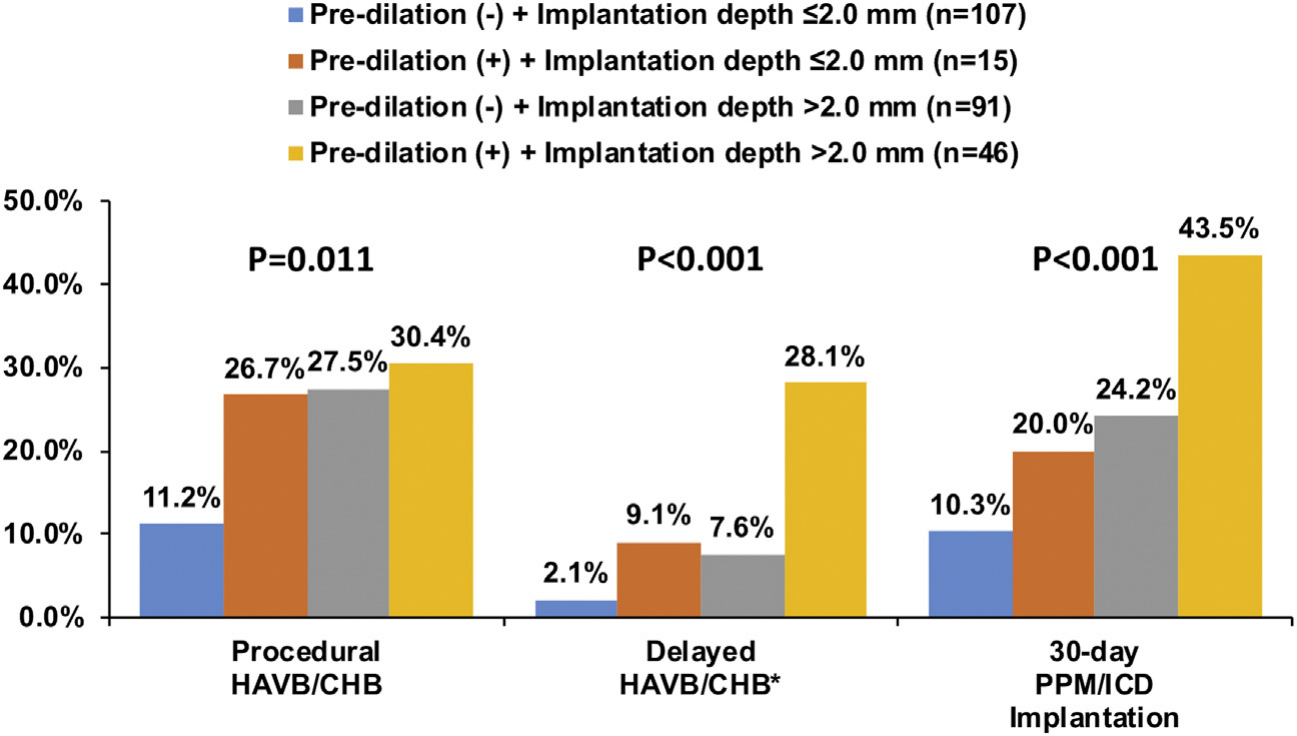
**Risk of 30-day HAVB/CHB and PPM/ICD requirement according to predilation and implantation depth.** This figure was made from the data of 259 patients with implantation depth available. ∗Includes 204 patients without procedural HAVB/CHB. Abbreviations: CHB, complete heart block; HAVB, high-degree atrioventricular block; ICD, implantable cardioverter defibrillator; PPM, permanent pacemaker.

The C-statistics of implantation depth for procedural HAVB/CHB, delayed HAVB/CHB, and 30-day PPM/ICD requirement were 0.63, 0.74, and 0.69, respectively (Supplemental Figure 3). Predilation and categorized implantation depth (>1.0 mm, >2.0 mm, or >3.0 mm) each showed a relatively high negative predictive value (>80%) in predicting these outcomes (particularly high at >95% for delayed HAVB/CHB; Supplemental Table 8). When predilation and categorized implantation depth were combined, the combination of predilation and implantation depth of >2.0 mm had a higher C-statistic for all the outcomes (procedural HAVB/CHB, delayed HAVB/CHB, and 30-day PPM) than the other combinations (Supplemental Figure 4). Therefore, for risk stratification, eligible patients were categorized into 4 groups according to predilation and implantation depth (>2.0 or ≤2.0 mm). Patients with no predilation and implantation depth of ≤2.0 mm had the lowest risk of HAVB/CHB and 30-day PPM/ICD requirement (Figure 3). Notably, delayed HAVB/CHB occurred in only 2.1% (2/95) of the patients with no predilation and implantation depth of ≤2.0 mm.

### Follow-Up Outcomes of Patients With or Without 30-Day PPM/ICD Requirement

**Figure 4:**
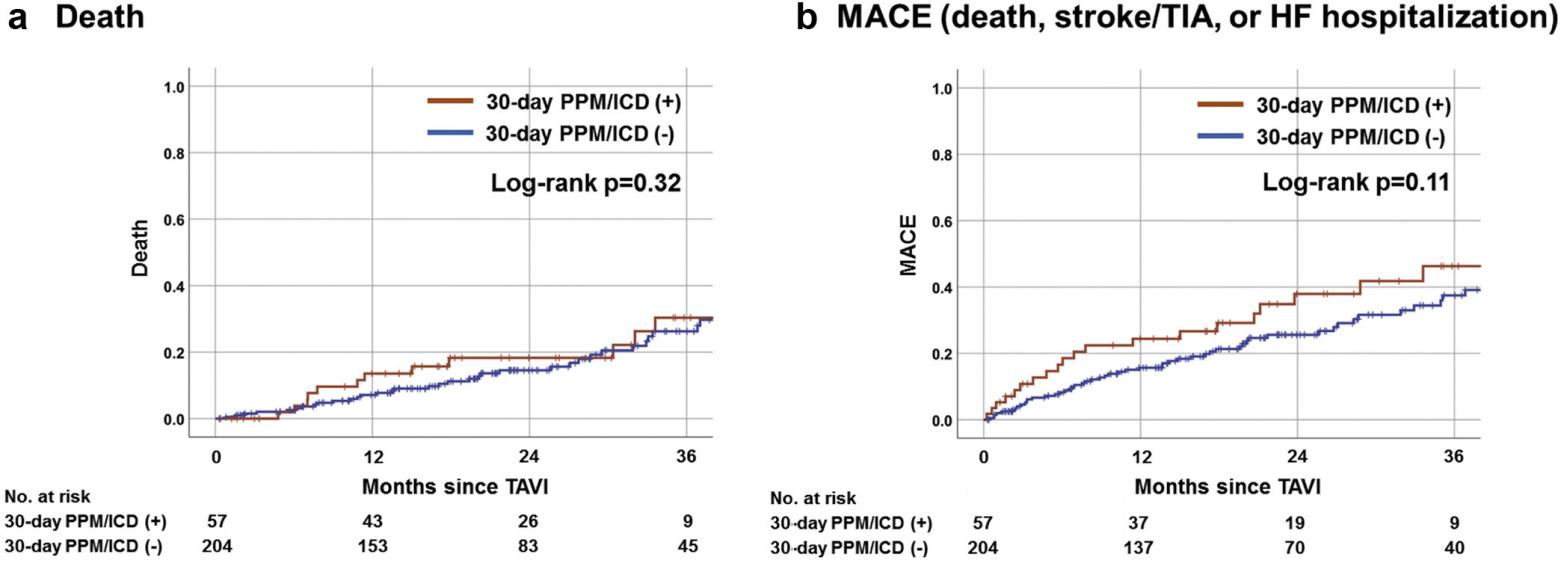
**Kaplan–Meier estimates of death and MACE of patients with or without 30-day PPM/ICD requirement.** This Figure shows the Kaplan–Meier curves with the log-rank test to compare (a) death and (b) MACE (death, stroke/TIA, or HF hospitalization) between patients with or without 30-day PPM/ICD among TAVI recipients with pre-existing RBBB. Abbreviations: HF, heart failure; ICD, implantable cardioverter defibrillator; MACE, major adverse cardiovascular events; PPM, permanent pacemaker; TIA, transient ischemic attack.

In the early phase (within 30 days after TAVI), no sudden death was observed; one patient died from TAVI-related stroke at a skilled nursing facility on day 25. During the median follow-up of 20.4 (IQR = 12.2-34.3) months, there was no significant difference between patients with or without the 30-day PPM/ICD with respect to death (26.3% vs. 18.6%; log-rank *p* = 0.32) and MACE (38.6% vs. 27.9%; log-rank *p* = 0.11) (Figure 4).

### Ventricular Pacing Rate After PPM/ICD Implantation

In 57 patients with the 30-day PPM/ICD, data on pacing rate at follow-up (median = 45 [IQR, 37, 61] days after PPM/ICD implantation) were available in 47 patients (40 dual-chamber PPMs; 1 single-chamber PPM; 1 leadless PPM; 5 CRT-P or CRT-D). In 42 patients with the PPM (excluding 5 patients with CRT-P or CRT-D), the median RV pacing rate was 78.5% (IQR = 18.0%-99.0%). Complete HAVB/CHB recovery (RV pacing < 1%) was observed in 3 (7.1%) patients, while PPM dependency (RV pacing > 40%) was observed in 28 (66.7%) patients.

## Discussion

The present study has several key findings: (1) the overall 30-day HAVB/CHB rate in pre-existing RBBB TAVI recipients was 28.0%, of which 76.7% occurred during TAVI procedure, followed by the same day of TAVI in 8.2% of individuals; (2) the delayed HAVB/CHB rate was 8.3% among patients without procedural HAVB/CHB, with a TPM reinsertion rate of 4.5%; (3) implantation depth independently predicted HAVB/CHB or/and 30-day PPM/ICD requirement; (4) a combination of no predilation and implantation depth of ≤2.0 mm harbored a relatively low risk of procedural HAVB/CHB (11.2%), delayed HAVB/CHB (2.1%), and PPM/ICD requirement (10.3%) at 30 days; (5) complete HAVB/CHB recovery after PPM implantation was uncommon.

### Incidence and Timing of HAVB/CHB Occurrence After TAVI

To date, many studies have examined the PPM risk in the overall TAVI population.^7^ However, only the present study and a recent Canadian study [n = 110]^22^ investigated the details of HAVB/CHB occurrence and subsequent need of the PPM among patients with pre-existing RBBB. Although the present study demonstrated a much lower HAVB/CHB rate than the Canadian study (28.0% vs. 55.5%), these 2 studies have several findings in common. First, the majority of HAVB/CHB events occurred during TAVI procedure (76.7% vs. 86.4%). Second, delayed HAVB/CHB occurred in an early period after TAVI (within 13 days vs. within 7 days) with a similar incidence (6.5% vs. 7.2%). These findings may suggest that despite different HAVB/CHB risks across clinical settings, HAVB/CHB mostly occurs periprocedurally, whereas delayed HAVB/CHB typically occurs in a very early phase with an incidence of ∼7%.

Timing of TPM removal remains controversial in patients with pre-existing RBBB. Recent expert consensus documents recommend maintaining the TPM for 24 h (or at least overnight) following TAVI in all patients with pre-existing RBBB.^2^^,^^3^ The present study revealed that the need for TPM reinsertion is relatively uncommon (4.5%) after TPM removal at the end of the TAVI procedure. This finding suggests that TPM removal immediately after TAVI followed by close telemetry monitoring with availability of rapid TPM reinsertion may be a reasonable strategy in many patients with pre-existing RBBB except for procedural HAVB/CHB cases.

### Additional Predictors of HAVB/CHB Among Patients With Pre-Existing RBBB

The prevalence of pre-existing RBBB in our TAVI recipients was 11.4%, comparable to other studies (5%-20%).^8^ Given the non-negligible prevalence and high post-TAVI PPM risk of pre-existing RBBB, additional factors for increased HAVB/CHB risk should be explored to help further risk stratification and facilitate safe yet timely discharge in patients with pre-existing RBBB. The aforementioned Canadian study investigated such factors in patients with pre-existing RBBB, reporting older age and pre-existing first degree atrioventricular block as predictors of increased PPM risk.^22^ However, implantation depth was not reported in that analysis. Recent studies reported implantation depth as a strong independent predictor of post-TAVI PPM risk,^7^^,^^23^^,^^24^ which has a sound anatomical basis.^4^ The present study revealed implantation depth as an independent predictor of procedural and delayed HAVB/CHB and 30-day PPM requirement among patients with pre-existing RBBB. In addition, predilation was a predictor of delayed HAVB/CHB. Importantly, unlike anatomical and electrical predisposing factors, these 2 procedural factors are potentially modifiable by operators to reduce the HAVB/CHB and PPM risks.

### Procedural Strategy and Risk Stratification

**Figure 5:**
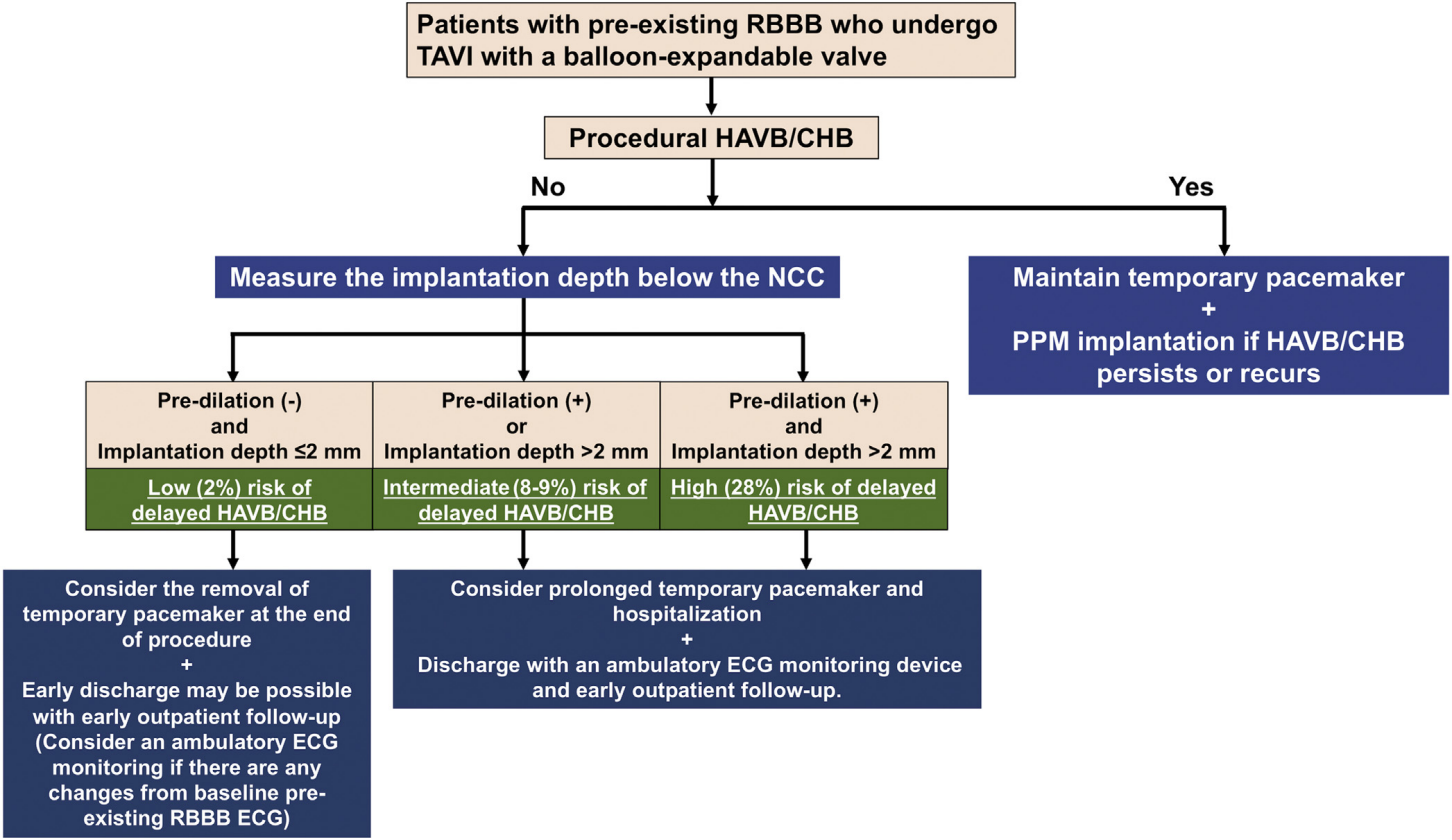
**Proposal for risk stratification using predilation and implantation depth among patients with pre-existing RBBB undergoing TAVI.** Abbreviations: CHB, complete heart block; ECG, electrocardiogram; HAVB, high-degree atrioventricular block; ICD, implantable cardioverter defibrillator; NCC, noncoronary cusp; RBBB, right bundle branch block; TAVI, transcatheter aortic valve implantation.

Minimizing the procedural HAVB/CHB risk and detecting delayed HAVB/CHB appropriately are essential goals in the conduction disorder management of TAVI recipients with pre-existing RBBB. The present results suggest that avoiding predilation and deploying THV at a higher position are important procedural strategies in this high PPM risk group. While procedural HAVB/CHB is easily detected by procedural monitoring, identification of delayed HAVB/CHB remains challenging. Delayed HAVB/CHB can potentially cause sudden death after discharge. Thus, risk stratification to identify the subpopulation at higher risk of delayed HAVB/CHB is clinically essential for early safe TPM removal and discharge in patients with pre-existing RBBB. Our data suggest that both predilation and categorized implantation depth have high negative predictive values for delayed HAVB/CHB (>95.0%). The combination of no predilation and implantation depth of ≤2.0 mm had a low risk (2.1%) of delayed HAVB/CHB, which may be helpful to select patients eligible for early safe TPM removal and discharge in the context of the recent trend toward a shorter hospital stay after TAVI.^5^^,^^25^ In the absence of procedural HAVB/CHB, TPM removal at the end of the TAVI procedure appears reasonable in patients with a balloon-expandable valve implanted at a higher position without predilation. Meanwhile, maintaining the TPM for 12-24 hours is also acceptable in inexperienced centers with less supportive infrastructure. In the other patients (especially those with both predilation and implantation depth of >2.0 mm), providing prolonged in-hospital observation and ambulatory ECG monitoring for at least 2 weeks should be considered in the early-phase management.^26^ We propose a risk stratification and subsequent management algorithm using predilation and implantation depth among patients with pre-existing RBBB undergoing TAVI (Figure 5).

One possible concern regarding a high valve deployment technique is the potential risk of valve migration or embolization. However, no valve migration or embolization occurred in the present study. In addition, our prior research demonstrates that while a high valve deployment technique led to a significantly lower rate of PPM implantation than conventional development (5.5% vs. 13.1%), there was only one valve embolization (0.2%) among 406 patients who underwent Sapien 3 TAVI with our high deployment technique,^16^ demonstrating the effectiveness and safety of the technique. Nonetheless, despite a wealth of experience in our center since the 2017 adoption of a higher valve deployment relative to the NCC isolated in the RAO caudal view as our standard TAVI technique for all types of THVs, a more formal replication across other centers of our technique would be helpful.

The present study was mainly limited to the Sapien valve during which our implantation technique evolved toward an NCC-based high implantation technique. As such, few patients received a self-expanding THV such as Evolut or ACURATE neo (Boston Scientific, Marlborough, Massachusetts); the ACURATE neo recently has been shown to associate with a lower 30-day PPM rate than Sapien 3 (29.6% vs. 43.9%, respectively) as reported by the SELECT RBBB (Transcatheter heart valve SELECTion in Patients with Right Bundle Branch Block) multicenter registry study,^27^ while the PPM rates in the study were globally much higher than in our cohort. Further studies are needed to better understand the impact of THV selection on the PPM risk in the context of high valve implantation.

### Follow-Up With or Without PPM Implantation

Our data found no significant difference in death during follow-up between patients with or without the 30-day PPM/ICD, which contradicts prior investigations reporting an increased cardiovascular mortality after TAVI in patients with pre-existing RBBB and without the PPM.^28^^,^^29^ Our negative result could be attributable partly to a small sample size. Thus, we should await a prospective multicenter study to determine the impact of the PPM in post-TAVI patients with pre-existing RBBB. Among patients requiring the PPM, the median RV pacing rate was as high as ∼80%, and complete HAVB/CHB recovery was uncommon (7.1%). These findings are consistent with prior observations^22^^,^^30^ and underscore the importance of PPM implantation in a timely fashion when necessary. Meanwhile, both the present and the Canadian studies^22^ demonstrate that patients with procedural transient HAVB/CHB may have a chance (50%-60%) to avoid PPM implantation. Since the determinants of persistent or transient HAVB/CHB are currently unknown, close follow-up and discussion with the electrophysiology team are important to judge the indications for the PPM.

### Study Limitations

The present study has several limitations to be acknowledged. This study was conducted in a single very high-volume U.S. center with the predominant use of balloon-expandable THVs and a unique high deployment technique based on the NCC basal plane. Therefore, the present findings may not be generalizable directly to other centers, especially those where the self-expanding THV is more frequently used with a conventional coplanar view deployment technique or in less experienced hands where valve migration/embolization may pose an issue. The number of self-expanding THV recipients is too small to assess the impact of the self-expanding THV on HAVB/CHB occurrence. The study’s retrospective design without routine ambulatory ECG monitoring may have precluded us from complete detection of asymptomatic arrhythmic events after discharge. The present study may be underpowered to examine the impact of PPM implantation on long-term outcomes owing to the small sample size of the PPM group. Finally, our risk stratification using predilation and implantation depth (Figure 5) requires validation in a prospective multicenter study.

## Conclusions

The present study found that 30-day HAVB/CHB occurred in 28.0% of patients with pre-existing RBBB (95% balloon-expandable THVs), with more than 3-quarters occurring during the TAVI procedure. Delayed HAVB/CHB was not rare, but TPM reinsertion was needed in only 4.5%, suggesting that early TPM removal may be possible in many patients. An absence of predilation coupled with an implantation depth of ≤2 mm portended the lowest overall 30-day PPM rate of 10.3% in our study patients, indicative of the importance of the procedural strategy, valve choice, and meticulous high implantation technique to potentially optimize outcomes in pre-existing RBBB TAVI recipients. Further prospective multicenter studies are required to establish appropriate management strategies (THV selection and deployment technique) in TAVI recipients with pre-existing RBBB.

## Ethics statement

The present study was approved by the Institutional Review Board of Cleveland Clinic with a waiver of informed consent owing to the retrospective nature of the study.

## Funding

This study was made possible by a generous gift from Jennifer and Robert McNeil. The funders had no role in the design and conduct of the study, in the collection, analysis, and interpretation of the data, and in the preparation, review, or approval of the manuscript.

## Disclosure statement

The authors declare no conflict of interest.
