# Supplementary material for: Risk Stratification and Management of Advanced Conduction Disturbances Following TAVI in Patients With Pre-Existing RBBB
Source: Struct Heart. 2022 Mar 17;6(1):100006. doi: 10.1016/j.shj.2022.100006 (PMC10236876; doi:10.1016/j.shj.2022.100006)
Supplement: Supplemental Figures 1-4 and Tables 1-8 [file mmc1.docx]

**Supplemental Figure 1. Patient selection**


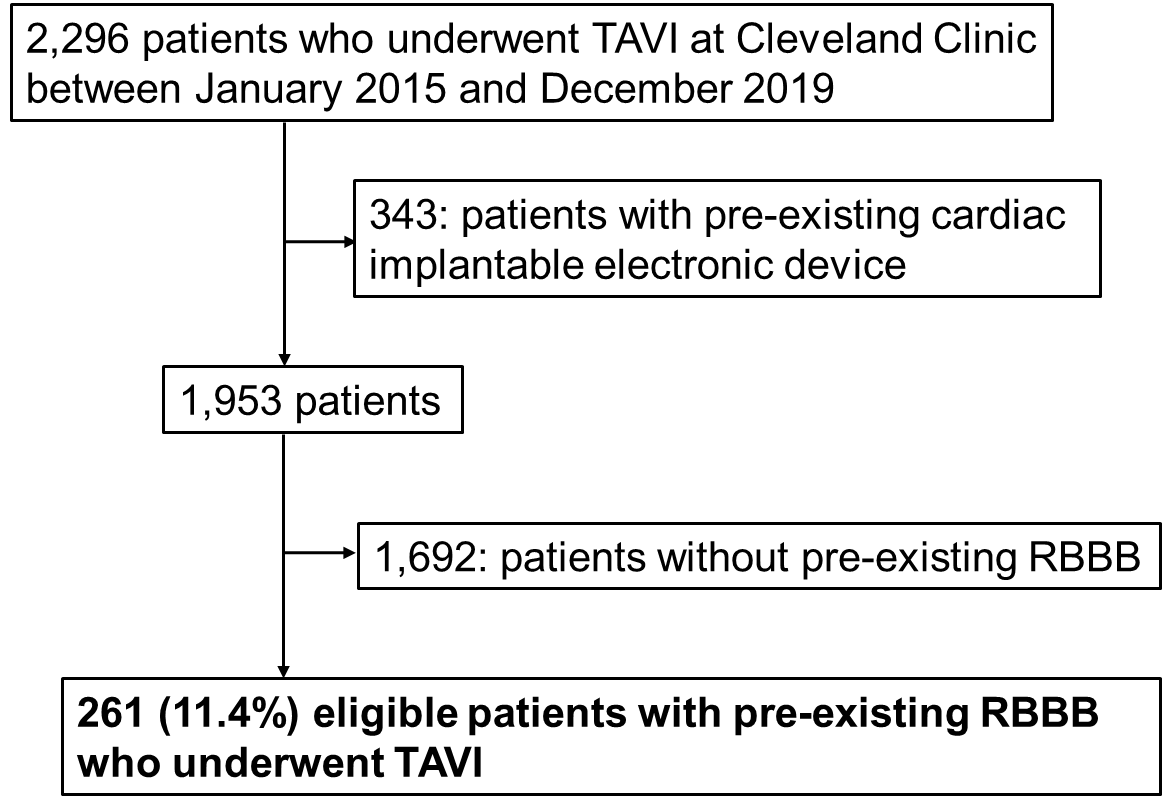


RBBB = right bundle branch block; TAVI = transcatheter aortic valve implantation.

**Supplemental Figure 2. Incidence of 30-day HAVB/CHB and PPM requirement post-TAVI with a balloon-expandable valve in patients with pre-existing RBBB**


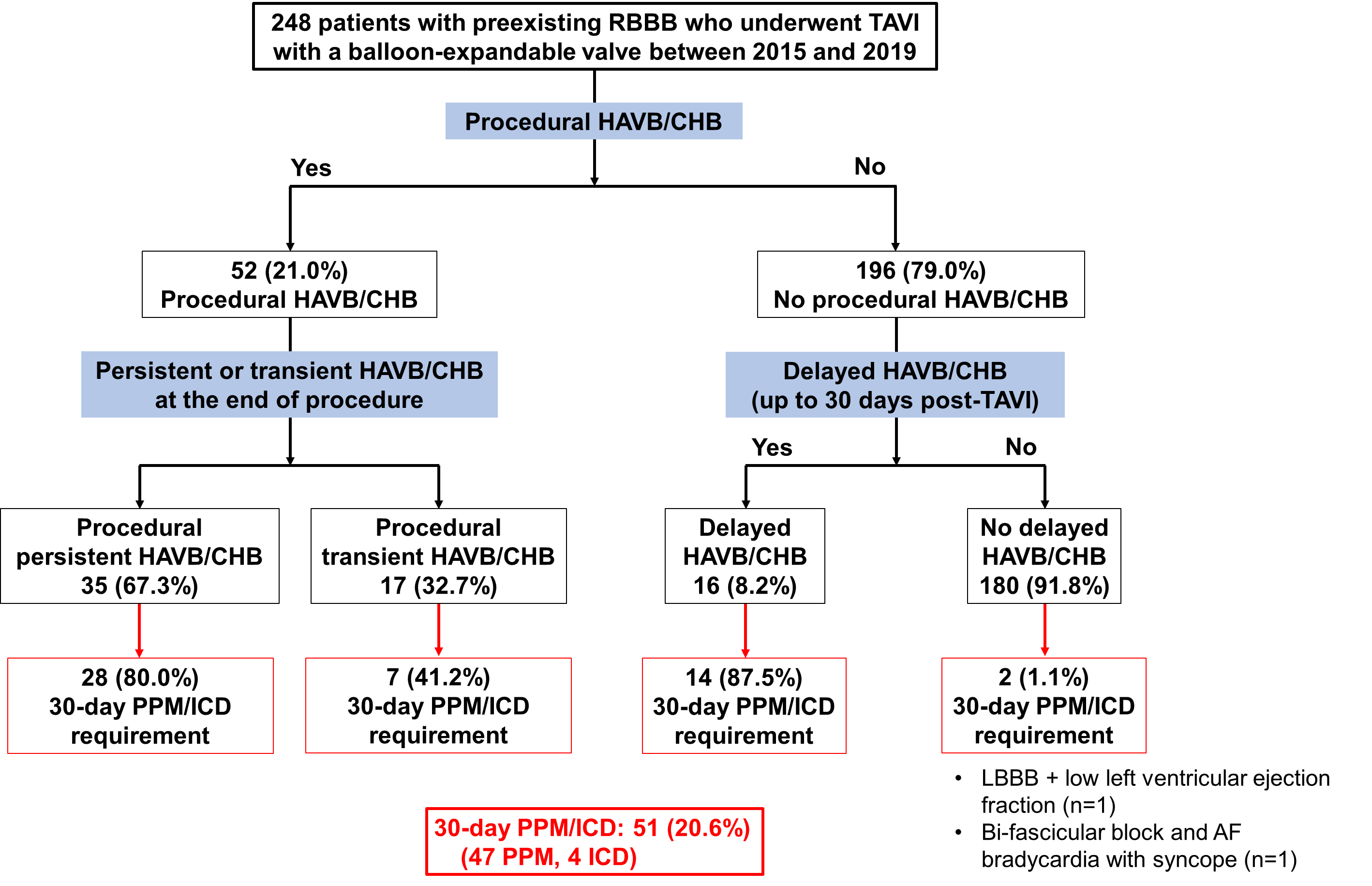


AF = atrial fibrillation; CHB = complete heart block; HAVB = high-degree atrioventricular block; ICD = implantable cardioverter defibrillator; LBBB = left bundle branch block; PPM = permanent pacemaker; RBBB = right bundle branch block; TAVI = transcatheter aortic valve implantation.

**Supplemental Figure 3. Receiver operating characteristic curve of implantation depth for HAVB/CHB and 30-day PPM/ICD requirement**


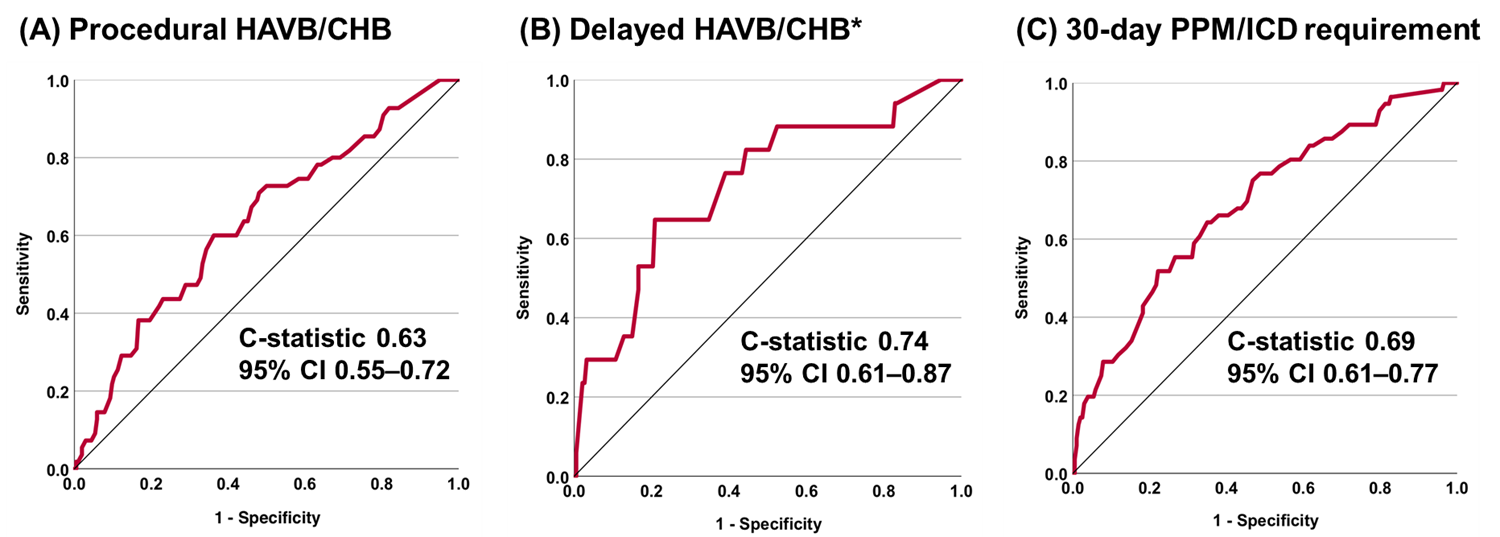


These figures were made from the data of 259 patients with implantation depth available. *Includes 204 patients without procedural HAVB/CHB. CI = confidence interval; other abbreviations as in Supplemental Figures 1 and 2.

**Supplemental Figure 4. Receiver operating characteristic curve for HAVB/CHB and 30-day PPM/ICD requirement by the combination of pre-dilation and implantation depth**


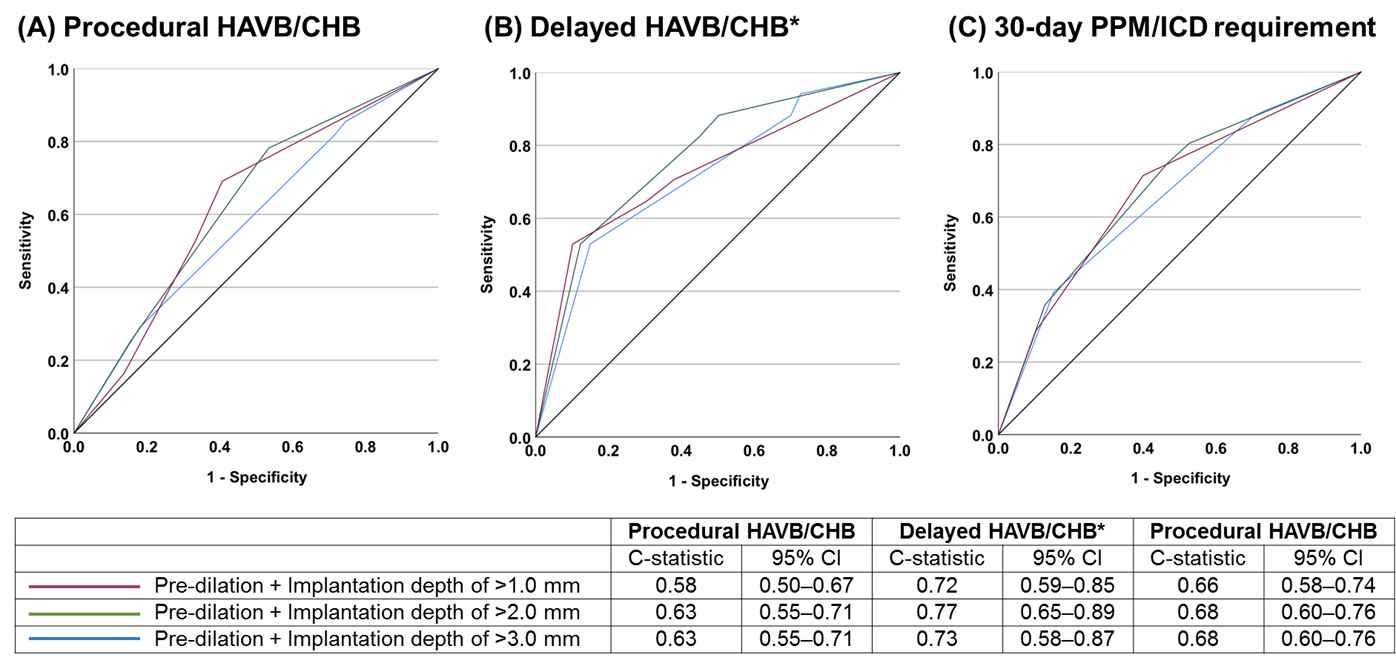


These figures were made from the data of 259 patients with implantation depth available. *Includes 204 patients without procedural HAVB/CHB. Abbreviations as in Supplemental Figures 1-3.

**Supplemental Table 1. Details of 9 patients who required TPM after TPM removed at the end of TAVI procedure in the absence of procedural HAVB/CHB**

| **Case No.** | **Age/sex** | **STS** **risk score (%)** | **Pre-TAVI PR/QRS duration (ms)** | **Immediately post-TAVI PR/QRS duration (ms)** | **Approach, diseased valve, and implanted prosthesis** | **Pre-/post-dilation** | **Implantation depth relative to NCC (mm)** | **Timing of TPM re-insertion post-TAVI** | **Reason for TPM re-insertion** | **Type of implanted device** |
| --- | --- | --- | --- | --- | --- | --- | --- | --- | --- | --- |
| **1** | 66M | 2.25 | 198/166 | 192/158 | Transaortic, native bicuspid, Sapien XT, 29mm | Yes/No | 6.1 | Day 3 | Symptomatic persistent CHB | Dual-chamber PPM |
| **2** | 73M | 1.37 | Atrial flutter  /158 | Atrial flutter  /161 | Transfemoral, native tricuspid, Sapien 3, 29mm | Yes/No | 6.8 | Same day of TAVI | Symptomatic persistent CHB | Dual-chamber PPM |
| **3** | 78M | 6.00 | AF/144 | AF/160 | Transfemoral, native tricuspid, Sapien 3, 29mm | Yes/No | 4.2 | Day 6 | Asymptomatic persistent CHB | Dual-chamber PPM |
| **4** | 79M | 5.38 | 216/152 | 218/144 | Transfemoral, native tricuspid, Sapien 3, 26mm | Yes/No | 4.2 | Day 1 | Asymptomatic intermittent CHB (->later persistent CHB) | Dual-chamber PPM |
| **5** | 84M | 4.65 | 156/164 | 154/160 | Transfemoral, native tricuspid, Sapien 3, 26mm | Yes/No | 6.0 | Day 1 | Asymptomatic intermittent CHB (->later persistent CHB) | Dual-chamber PPM |
| **6** | 87M | 4.29 | AF/122 | AF/126 | Transfemoral, native tricuspid, Sapien 3, 26mm | Yes/Yes | 3.7 | Same day of TAVI | Symptomatic persistent CHB | Single-chamber PPM |
| **7** | 81M | 1.92 | 194/130 | 278/162 | Transfemoral, native tricuspid, Sapien 3, 29mm | No/No | 5.8 | Same day of TAVI | Asymptomatic Mobitz type II 2:1 AVB with alternating bundle branch block | Dual-chamber PPM |
| **8** | 89M | 4.18 | 234/126 | 204/120 | Transfemoral, native tricuspid, Sapien 3, 26mm | No/No | 3.7 | Day 5 | Asymptomatic transient CHB | Dual-chamber PPM |
| **9** | 83M | 5.80 | AF/128 | AF/130 | Transfemoral, native tricuspid, Sapien 3, 26mm | No/No | 1.8 | Same day of TAVI | Asymptomatic persistent CHB | Dual-chamber PPM |

AF = atrial fibrillation; AVB = atrioventricular block; CHB = complete heart block; HAVB = high-degree atrioventricular block; NCC = non-coronary cusp; PPM = permanent pacemaker; STS = Society of Thoracic Surgeons; TAVI = transcatheter aortic valve implantation; TPM = temporary pacemaker.

**Supplemental Table 2. Baseline and procedural characteristics of patients with procedural persistent vs. procedural transient HAVB/CHB**

|  | **Procedural HAVB/CHB** | |  |
| --- | --- | --- | --- |
|  | **Procedural persistent (n=39)** | **Procedural transient (n=17)** | **P value** |
| Age, years | 85 (80.5–8.8.0) | 80.0 (73.0–86.0) | 0.090 |
| Female | 15 (38.5) | 6 (35.3) | 1.00 |
| Caucasian | 39 (100.0) | 15 (88.2) | 0.088 |
| Body mass index, kg/m^2^ | 27.2 (25.4–31.4) | 29.3 (26.0–36.7) | 0.28 |
| STS risk score, % | 6.86 (4.42–11.22) | 5.3 (4.36–7.75) | 0.34 |
| Prior CABG | 10 (25.6) | 4 (23.5) | 1.00 |
| Prior myocardial infarction | 9 (23.1) | 7 (41.2) | 0.21 |
| ESRD on dialysis | 4 (10.3) | 1 (5.9) | 1.00 |
| Chronic lung disease | 20 (51.3) | 11 (64.7) | 0.40 |
| History of syncope | 4 (10.3) | 1 (5.9) | 1.00 |
| History of atrial fibrillation/flutter | 18 (46.2) | 9 (52.9) | 0.77 |
| NYHA functional class III or IV | 33 (84.6) | 14 (82.4) | 1.00 |
| LVEF, % | 57 (51.5–63) | 55 (47–59) | 0.12 |
| Aortic valve area, cm^2^ | 0.64 (0.50–0.77) | 0.63 (0.59–0.78) | 0.78 |
| Aortic valve mean gradient, mmHg | 42 (36–53.5) | 48 (35–57) | 0.65 |
| Aortic valve peak gradient, mmHg | 69 (60.5–86.5) | 81 (55–90) | 0.84 |
| Bicuspid aortic valve | 1 (2.6) | 3 (17.6) | 0.079 |
| Failed bioprosthetic valve | 1 (2.6) | 0 (0.0) | 1.00 |
| Moderate or severe AR | 7 (17.9) | 2 (11.8) | 0.71 |
| Data on aortic annulus |  |  |  |
| Maximum annular diameter, mm | 28 (26.5–30) | 28 (25–31) | 0.84 |
| Minimum annular diameter, mm | 23 (21–25) | 22 (20–24) | 0.36 |
| Eccentricity index | 0.19 (0.15–0.25) | 0.21 (0.16–0.27) | 0.63 |
| Annular area, mm^2^ | 497 (416–560) | 460 (410–546) | 0.57 |
| Calcium score of aortic valve leaflets, HU | 2099 (1535–3293) [n=32] | 1338 (1036–3418) [n=16] | 0.25 |
| LVOT calcification | 29/38 (76.3) | 8/17 (47.1) | 0.060 |
| **Pre-TAVI baseline ECG findings** |  |  |  |
| Rhythm |  |  | 0.72 |
| Sinus rhythm | 30 (76.9) | 15 (88.2) |  |
| Atrial fibrillation | 7 (17.9) | 2 (11.8) |  |
| Atrial flutter | 1 (2.6) | 0 (0.0) |  |
| Junctional rhythm | 1 (2.6) | 0 (0.0) |  |
| PR interval, ms | 185 (172–220) [n=30] | 212 (176–250) [n=15] | 0.20 |
| QRS duration, ms | 144 (137–152) | 148 (138–154) | 0.89 |
| 1st degree AVB | 10 (25.6) | 9 (52.9) | 0.067 |
| QRS ≥150 ms | 16 (41.0) | 6 (35.3) | 0.77 |
| Left anterior fascicular block | 9 (23.1) | 4 (23.5) | 1.00 |
| Left posterior fascicular block | 0 (0.0) | 0 (0.0) | (-) |
| Bi-fascicular block | 9 (23.1) | 4 (23.5) | 1.00 |
| Tri-fascicular block | 3 (7.7) | 2 (11.8) | 0.63 |
| **Procedural details** |  |  |  |
| Non-elective procedure | 1 (2.6) | 1 (5.9) | 0.52 |
| Non-femoral approach | 4 (10.3) | 1 (5.9) | 1.00 |
| Type of anesthesia |  |  | 1.00 |
| Conscious sedation | 30 (76.9) | 13 (76.5) |  |
| General anesthesia | 9 (23.1) | 4 (23.5) |  |
| Valve type |  |  | 0.30 |
| Balloon-expandable | 35 (89.7) | 17 (100.0) |  |
| Self-expanding | 4 (10.3) | 0 (0.0) |  |
| Valve size |  |  | 0.59 |
| ≤23 mm | 11 (28.2) | 7 (41.2) |  |
| 26 mm | 12 (30.8) | 5 (29.4) |  |
| ≥29 mm | 16 (41.0) | 5 (29.4) |  |
| Pre-dilation | 13 (33.3) | 5 (29.4) | 1.00 |
| Post-dilation | 22 (56.4) | 5 (29.4) | 0.084 |
| Oversizing, % | 7.1 (2.4–9.8) | 3.7 (-0.5–7.4) | 0.21 |
| Implantation depth relative to NCC, mm | 3.5 (1.5–5.2) [n=39] | 3.0 (1.9–4.6) [n=16] | 0.51 |
| **In-hospital adverse events** |  |  |  |
| Death | 0 (0.0) | 0 (0.0) | (-) |
| Major vascular complication | 1 (2.6) | 0 (0.0) | 1.00 |
| Conversion to open surgery | 0 (0.0) | 0 (0.0) | (-) |
| Coronary obstruction | 0 (0.0) | 0 (0.0) | (-) |
| 2nd valve deployment | 5 (12.8) | 0 (0.0) | 0.31 |
| New-onset atrial fibrillation | 2 (5.1) | 0 (0.0) | 1.00 |
| Stroke/transient ischemic attack | 1 (2.6) | 1 (5.9) | 0.52 |
| Overall length of stay, days | 4 (3–9) | 4 (3–5) | 0.51 |
| Post-TAVI length of stay, days | 4 (3–8.5) | 4 (3–5) | 0.37 |

Values are n (%), n/total n (%), or median (interquartile range). AR = aortic regurgitation; AV = aortic valve; AVB = atrioventricular block; CABG = coronary artery bypass grafting; CHB = complete heart block; CT = computed tomography; ECG = electrocardiogram; HAVB = high-degree atrioventricular block; HU = Hounsfield unit; ESRD = end-stage renal disease; LVEF = left ventricular ejection fraction; LVOT = left ventricular outflow tract; NCC = non-coronary cusp; NYHA = New York Heart Association; STS = Society of Thoracic Surgeons; TAVI = transcatheter aortic valve implantation.

**Supplemental Table 3. Baseline and procedural characteristics of patients who did and did not require PPM/ICD within 30 days post-TAVI**

|  | **30-day PPM/ICD requirement** | |  |
| --- | --- | --- | --- |
|  | **No (n=204)** | **Yes (n=57)** | **P value** |
| Age, years | 81 (76–86) | 83 (76–87) | 0.43 |
| Female | 57 (27.9) | 16 (28.1) | 1.00 |
| Caucasian | 194 (95.1) | 57 (100.0) | 0.12 |
| Body mass index, kg/m^2^ | 29.3 (24.9–33.5) | 28.1 (26.3–33.2) | 0.89 |
| STS risk score, % | 4.97 (3.41–8.04) | 5.38 (4.03–9.39) | 0.31 |
| Prior CABG | 66 (32.4) | 17 (29.8) | 0.75 |
| Prior myocardial infarction | 47 (23.0) | 16 (28.1) | 0.48 |
| ESRD on dialysis | 7 (3.4) | 5 (8.8) | 0.14 |
| Chronic lung disease | 103 (50.5) | 29 (50.9) | 1.00 |
| History of syncope | 11 (5.4) | 7 (12.3) | 0.080 |
| History of atrial fibrillation/flutter | 76 (37.3) | 25 (43.9) | 0.44 |
| NYHA functional class III or IV | 162 (79.4) | 48 (84.2) | 0.57 |
| LVEF, % | 59 (54.5–64) | 57 (52–63) | 0.44 |
| Aortic valve area, cm^2^ | 0.72 (0.60–0.84) [n=188] | 0.66 (0.55–0.80) [n=57] | 0.084 |
| Aortic valve mean gradient, mmHg | 41 (33.5–51) | 42 (34–55) | 0.72 |
| Aortic valve peak gradient, mmHg | 70 (58–85) | 70 (58–88) | 0.82 |
| Bicuspid aortic valve | 10 (4.9) | 4 (7.0) | 0.51 |
| Failed bioprosthetic valve | 17 (8.3) | 1 (1.8) | 0.13 |
| Moderate or severe AR | 37 (18.1) | 11 (19.3) | 0.85 |
| Data on aortic annulus | [n=196] | [n=57] |  |
| Maximum annular diameter, mm | 28 (26–30) | 28 (27–30) | 0.51 |
| Minimum annular diameter, mm | 23 (21–24.9) | 23 (21–25) | 0.80 |
| Eccentricity index | 0.18 (0.14–0.23) | 0.19 (0.15–0.25) | 0.16 |
| Annular area, mm^2^ | 493 (411–565.5) | 497 (430–560) | 0.95 |
| Calcium score of aortic valve leaflets, HU | 2192 (1255–3261) [n=149] | 2051 (1332–3293) [n=48] | 0.63 |
| LVOT calcification | 95/185 (51.4) | 40/56 (71.4) | 0.009 |
| **Pre-TAVI baseline ECG findings** |  |  |  |
| Rhythm |  |  | 0.72 |
| Sinus rhythm | 173 (84.8) | 45 (78.9) |  |
| Atrial fibrillation | 25 (12.3) | 10 (17.5) |  |
| Atrial flutter | 4 (2.0) | 1 (1.8) |  |
| Junctional rhythm | 2 (1.0) | 1 (1.8) |  |
| PR interval, ms | 188 (169–220) [n=176] | 194 (173–220) [n=44] | 0.59 |
| QRS duration, ms | 146 (136–156) | 148 (138–154) | 0.84 |
| 1st degree AVB | 69 (33.8) | 18 (31.6) | 0.87 |
| QRS duration ≥150 ms | 81 (39.7) | 28 (49.1) | 0.23 |
| Left anterior fascicular block | 55 (27.0) | 15 (26.3) | 1.00 |
| Left posterior fascicular block | 2 (1.0) | 1 (1.8) | 0.52 |
| Bi-fascicular block | 57 (27.9) | 16 (28.1) | 1.00 |
| Tri-fascicular block | 18 (8.8) | 6 (10.5) | 0.80 |
| **Procedural details** |  |  |  |
| Non-elective procedure | 9 (4.4) | 3 (5.3) | 0.73 |
| Non-femoral approach | 12 (5.9) | 5 (8.8) | 0.54 |
| Type of anesthesia |  |  | 0.075 |
| Conscious sedation | 173 (84.8) | 42 (73.7) |  |
| General anesthesia | 31 (15.2) | 15 (26.3) |  |
| Valve type |  |  | 0.041 |
| Balloon-expandable | 197 (96.6) | 51 (89.5) |  |
| Self-expanding | 7 (3.4) | 6 (10.5) |  |
| Valve size |  |  | 0.65 |
| ≤23 mm | 59 (28.9) | 14 (24.6) |  |
| 26 mm | 79 (38.7) | 21 (36.8) |  |
| ≥29 mm | 66 (32.4) | 22 (38.6) |  |
| Pre-dilation | 38 (18.6) | 24 (42.1) | <0.001 |
| Post-dilation | 91 (44.6) | 27 (47.4) | 0.76 |
| Oversizing, % | 4.6 (0.7–7.7) [n=196] | 6.3 (2.5–9.8) [n=57] | 0.044 |
| Implantation depth relative to NCC, mm | 1.9 (0.8–3.6)  [n=203] | 3.7 (2.1–5.3)  [n=56] | <0.001 |
| **In-hospital adverse events** |  |  |  |
| In-hospital death | 0 (0.0) | 0 (0.0) | (-) |
| Major vascular complication | 0 (0.0) | 1 (1.8) | 0.22 |
| Conversion to open surgery | 0 (0.0) | 0 (0.0) | (-) |
| Coronary obstruction | 1 (0.5) | 0 (0.0) | 1.00 |
| 2nd valve deployment | 0 (0.0) | 5 (8.8) | <0.001 |
| New-onset atrial fibrillation | 4 (2.0) | 4 (7.0) | 0.072 |
| Paravalvular leak ≥2+ | 2 (1.0) | 1 (1.8) | 0.52 |
| Stroke/transient ischemic attack | 3 (1.5) | 2 (3.5) | 0.30 |
| Overall length of stay | 2 (1–4) | 4 (3–8) | <0.001 |
| Post-TAVI length of stay | 2 (1–3) | 4 (3–8) | <0.001 |

Values are n (%), n/total n (%), or median (interquartile range). ICD = implantable cardioverter defibrillator; PPM = permanent pacemaker; other abbreviations as in Supplemental Table 2.

**Supplemental Table 4. Univariable logistic regression analyses for procedural HAVB/CHB, delayed HAVB/CHB, and 30-day PPM/ICD requirement.**

|  | **Procedural HAVB/CHB** | | | **Delayed HAVB/CHB** | | | **30-day PPM/ICD requirement** | | |
| --- | --- | --- | --- | --- | --- | --- | --- | --- | --- |
|  | **OR** | **95% CI** | **P value** | **OR** | **95% CI** | **P value** | **OR** | **95% CI** | **P value** |
| Age, per 1 year increase | 1.02 | 0.99–1.06 | 0.24 | 1.01 | 0.95–1.06 | 0.85 | 1.01 | 0.98–1.05 | 0.43 |
| Female | 1.77 | 0.94–3.30 | 0.075 | 0.61 | 0.17–2.21 | 0.45 | 1.01 | 0.52–1.93 | 0.98 |
| STS risk score, per 1% increase | 1.09 | 1.02–1.16 | **0.008** | 0.90 | 0.76–1.07 | 0.22 | 1.04 | 0.98–1.11 | 0.19 |
| Prior CABG | 0.66 | 0.34–1.28 | 0.22 | 1.08 | 0.38–3.06 | 0.88 | 0.89 | 0.47–1.68 | 0.72 |
| Prior myocardial infarction | 1.34 | 0.69–2.61 | 0.38 | 0.70 | 0.19–2.55 | 0.59 | 1.30 | 0.67–2.53 | 0.43 |
| History of syncope | 1.45 | 0.49–4.25 | 0.50 | 3.81 | 0.94–15.47 | 0.061 | 2.46 | 0.91–6.66 | 0.077 |
| History of atrial fibrillation/flutter | 1.65 | 0.91–2.99 | 0.10 | 1.64 | 0.61–4.46 | 0.33 | 1.32 | 0.73–2.39 | 0.37 |
| NYHA functional class III or IV | 1.35 | 0.61–2.96 | 0.46 | 0.82 | 0.25–2.67 | 0.75 | 1.38 | 0.63–3.04 | 0.42 |
| LVEF, per 1% increase | 0.98 | 0.95–1.01 | 0.12 | 1.01 | 0.96–1.06 | 0.71 | 0.99 | 0.97–1.02 | 0.54 |
| Aortic valve area, per 0.1 cm^2^ decrease | 1.27 | 1.07–1.52 | **0.008** | 0.99 | 0.75–1.32 | 0.97 | 1.15 | 0.97–1.36 | 0.11 |
| Aortic valve mean gradient, per 1 mmHg increase | 1.01 | 0.99–1.03 | 0.40 | 1.00 | 0.96–1.03 | 0.84 | 1.00 | 0.99–1.02 | 0.66 |
| Bicuspid aortic valve | 1.50 | 0.45–4.98 | 0.51 | 1.24 | 0.15–10.44 | 0.84 | 1.46 | 0.44–4.85 | 0.53 |
| Failed bioprosthetic valve | 0.20 | 0.03–1.54 | 0.12 | – | – | – | 0.20 | 0.03–1.51 | 0.12 |
| Moderate or severe AR | 0.82 | 0.37–1.80 | 0.61 | 0.90 | 0.25–3.32 | 0.88 | 1.08 | 0.51–2.28 | 0.84 |
| Eccentricity index, per 0.01 increase | 1.02 | 0.98–1.06 | 0.31 | 1.03 | 0.97–1.10 | 0.39 | 1.02 | 0.98–1.06 | 0.34 |
| Annular area, per 10 mm^2^ increase | 0.99 | 0.96–1.02 | 0.65 | 1.01 | 0.96–1.06 | 0.80 | 1.00 | 0.97–1.03 | 0.99 |
| Calcium score of aortic valve leaflets, per 100 HU increase | 1.01 | 0.98–1.03 | 0.63 | 0.99 | 0.95–1.04 | 0.78 | 1.01 | 0.98–1.03 | 0.46 |
| LVOT calcification | 1.85 | 0.98–3.47 | 0.058 | 1.01 | 0.37–2.75 | 0.98 | 2.37 | 1.24–4.53 | **0.009** |
| Non-sinus rhythm | 1.32 | 0.62–2.82 | 0.47 | 2.48 | 0.81–7.62 | 0.11 | 1.49 | 0.71–3.13 | 0.29 |
| PR interval, per 10ms increase | 1.02 | 0.94–1.10 | 0.65 | 1.10 | 0.97–1.25 | 0.15 | 1.02 | 0.94–1.11 | 0.59 |
| QRS duration, per 10ms increase | 0.98 | 0.81–1.19 | 0.84 | 0.84 | 0.6–1.17 | 0.30 | 1.02 | 0.85–1.23 | 0.84 |
| 1st degree AVB | 1.03 | 0.55–1.93 | 0.92 | 1.11 | 0.39–3.14 | 0.85 | 0.90 | 0.48–1.69 | 0.75 |
| QRS duration ≥150 ms | 0.88 | 0.48–1.60 | 0.67 | 1.23 | 0.45–3.32 | 0.69 | 1.47 | 0.81–2.65 | 0.20 |
| Bi-fascicular block | 0.73 | 0.37–1.46 | 0.37 | 1.01 | 0.34–3.00 | 0.99 | 1.01 | 0.52–1.93 | 0.98 |
| Tri-fascicular block | 0.96 | 0.34–2.7 | 0.94 | 1.34 | 0.28–6.37 | 0.71 | 1.22 | 0.46–3.22 | 0.69 |
| Non-elective procedure | 0.72 | 0.15–3.40 | 0.68 | 3.00 | 0.58–15.41 | 0.19 | 1.20 | 0.31–4.60 | 0.79 |
| Non-femoral approach | 1.58 | 0.53–4.68 | 0.41 | 1.01 | 0.12–8.30 | 1.00 | 1.54 | 0.52–4.56 | 0.44 |
| General anesthesia (vs. conscious sedation) | 1.58 | 0.76–3.25 | 0.22 | 3.25 | 1.11–9.53 | **0.032** | 1.99 | 0.99–4.02 | 0.054 |
| Balloon-expandable valve (vs. Self-expanding valve) | 1.68 | 0.50–5.66 | 0.41 | 1.41 | 0.17–11.96 | 0.75 | 3.31 | 1.07–10.28 | **0.038** |
| Valve size: ≥29 mm (vs. ≤23 mm) | 0.63 | 0.30–1.32 | 0.22 | 7.40 | 0.92–59.54 | 0.060 | 1.12 | 0.53–2.39 | 0.77 |
| Valve size: 26 mm (vs. ≤23 mm) | 0.96 | 0.46–1.97 | 0.91 | 5.31 | 0.62–45.53 | 0.13 | 1.40 | 0.66–2.99 | 0.38 |
| Pre-dilation | 1.73 | 0.90–3.33 | 0.098 | 6.47 | 2.39–18.21 | **<0.001** | 3.18 | 1.69–5.98 | **<0.001** |
| Post-dilation | 1.17 | 0.65–2.11 | 0.61 | 0.49 | 0.17–1.46 | 0.20 | 1.12 | 0.62–2.01 | 0.71 |
| Oversizing, %, per 1% increase | 1.00 | 0.99–1.02 | 0.81 | 1.00 | 0.98–1.03 | 0.79 | 1.00 | 0.99–1.02 | 0.58 |
| Implantation depth relative to NCC, per 1mm increase | 1.27 | 1.09–1.48 | **0.002** | 1.53 | 1.18–1.99 | **0.001** | 1.42 | 1.21–1.66 | **<0.001** |

CI = confidence interval; ICD = implantable cardioverter defibrillator; OR = odds ratio; PPM = permanent pacemaker; RBBB = right bundle branch block; other abbreviations as in Supplemental Table 2.

**Supplemental Table 5. Complete-case multivariable analyses for predictors of HAVB/CHB and 30-day PPM/ICD post-TAVI in patient with pre-existing RBBB**

| **Procedural HAVB/CHB (n=243)** | **OR** | **95% CI** | **P value** |
| --- | --- | --- | --- |
| STS risk score, per 1% increase | 1.09 | 1.02–1.16 | 0.016 |
| Aortic valve area, per 0.1 cm^2^ decrease | 1.24 | 1.03–1.49 | 0.023 |
| Implantation depth relative to NCC, per 1 mm increase | 1.28 | 1.09–1.50 | 0.003 |
| **Delayed HAVB/CHB (n=204)*** |  |  |  |
| General anesthesia (vs. conscious sedation) | 1.00 | 0.28–3.57 | 1.00 |
| Pre-dilation | 4.18 | 1.26–13.85 | 0.019 |
| Implantation depth relative to NCC, per 1 mm increase | 1.34 | 1.01–1.79 | 0.044 |
| **30-day PPM/ICD requirement (n=239)** |  |  |  |
| LVOT calcification | 1.71 | 0.85–3.42 | 0.13 |
| Self-expanding valve (vs. balloon-expandable valve) | 1.91 | 0.40–9.11 | 0.42 |
| Pre-dilation | 1.78 | 0.88–3.62 | 0.11 |
| Implantation depth relative to NCC, per 1 mm increase | 1.34 | 1.13–1.59 | 0.001 |

Predictors were examined in multivariable logistic regression models using variables with p value at <0.05 in univariable models (**Supplemental Table 3**). *Includes patients without procedural HAVB/CHB. Abbreviations as in Supplemental Tables 2-4.

**Supplemental Table 6. Univariable logistic regression analyses for procedural HAVB/CHB, delayed HAVB/CHB, and 30-day PPM/ICD requirement post-TAVI with a balloon-expandable valve**

|  | **Procedural HAVB/CHB** | | | **Delayed HAVB/CHB** | | | **30-day PPM/ICD requirement** | | |
| --- | --- | --- | --- | --- | --- | --- | --- | --- | --- |
|  | **OR** | **95% CI** | **P value** | **OR** | **95% CI** | **P value** | **OR** | **95% CI** | **P value** |
| Age, per 1 year increase | 1.02 | 0.98–1.06 | 0.33 | 1.01 | 0.95–1.08 | 0.68 | 1.02 | 0.98–1.05 | 0.39 |
| Female | 1.78 | 0.93–3.41 | 0.084 | 0.42 | 0.09–1.90 | 0.26 | 0.91 | 0.45–1.83 | 0.78 |
| STS risk score, per 1% increase | 1.09 | 1.02–1.16 | **0.012** | 0.91 | 0.76–1.08 | 0.27 | 1.04 | 0.97–1.11 | 0.32 |
| Prior CABG | 1.58 | 0.80–3.12 | 0.19 | 0.48 | 0.11–2.22 | 0.35 | 1.28 | 0.64–2.57 | 0.49 |
| Prior myocardial infarction | 0.69 | 0.34–1.38 | 0.29 | 1.26 | 0.44–3.64 | 0.67 | 0.91 | 0.46–1.78 | 0.78 |
| History of syncope | 1.17 | 0.37–3.76 | 0.79 | 3.92 | 0.96–16.04 | 0.057 | 2.25 | 0.79–6.42 | 0.13 |
| History of atrial fibrillation/flutter | 1.63 | 0.88–3.02 | 0.12 | 1.86 | 0.67–5.18 | 0.24 | 1.40 | 0.75–2.60 | 0.29 |
| NYHA functional class III or IV | 1.23 | 0.55–2.72 | 0.62 | 1.12 | 0.30–4.14 | 0.86 | 1.41 | 0.62–3.24 | 0.41 |
| LVEF, per 1% increase | 0.98 | 0.95–1.00 | 0.11 | 1.02 | 0.96–1.07 | 0.58 | 0.99 | 0.96–1.02 | 0.51 |
| Aortic valve area, per 0.1 cm^2^ decrease | 1.24 | 1.03–1.48 | **0.022** | 1.00 | 0.75–1.34 | 0.99 | 1.14 | 0.96–1.36 | 0.14 |
| Aortic valve mean gradient, per 1 mmHg increase | 1.00 | 0.98–1.02 | 0.80 | 0.99 | 0.96–1.03 | 0.72 | 1.00 | 0.98–1.02 | 0.88 |
| Bicuspid aortic valve | 1.55 | 0.47–5.16 | 0.47 | 1.27 | 0.15–10.68 | 0.83 | 1.59 | 0.48–5.30 | 0.45 |
| Failed bioprosthetic valve | – | – | – | – | – | – | – | – | – |
| Moderate or severe AR | 0.93 | 0.42–2.08 | 0.86 | 1.03 | 0.28–3.81 | 0.97 | 1.32 | 0.61–2.83 | 0.48 |
| Eccentricity index, per 0.01 increase | 1.02 | 0.98–1.06 | 0.38 | 1.01 | 0.95–1.09 | 0.71 | 1.01 | 0.97–1.05 | 0.62 |
| Annular area, per 10 mm^2^ increase | 1.00 | 0.97–1.03 | 0.78 | 1.01 | 0.96–1.06 | 0.75 | 1.00 | 0.97–1.03 | 0.92 |
| Calcium score of aortic valve leaflets, per 100 HU increase | 1.00 | 0.98–1.03 | 0.82 | 0.99 | 0.95–1.04 | 0.73 | 1.01 | 0.98–1.03 | 0.65 |
| LVOT calcification | 1.77 | 0.93–3.36 | 0.082 | 0.93 | 0.33–2.59 | 0.89 | 2.12 | 1.09–4.09 | **0.026** |
| Non-sinus rhythm | 1.11 | 0.49–2.52 | 0.79 | 2.69 | 0.86–8.38 | 0.087 | 1.36 | 0.61–3.00 | 0.45 |
| PR interval, ms | 1.01 | 0.93–1.10 | 0.74 | 1.11 | 0.97–1.27 | 0.14 | 1.03 | 0.95–1.12 | 0.52 |
| QRS duration, ms | 0.97 | 0.80–1.18 | 0.78 | 0.82 | 0.58–1.16 | 0.27 | 1.01 | 0.83–1.23 | 0.91 |
| 1st degree AVB | 1.07 | 0.56–2.03 | 0.84 | 0.91 | 0.30–2.74 | 0.87 | 0.89 | 0.46–1.72 | 0.72 |
| QRS duration ≥150 ms | 0.80 | 0.43–1.49 | 0.48 | 1.31 | 0.47–3.64 | 0.61 | 1.38 | 0.74–2.55 | 0.31 |
| Bi-fascicular block | 0.85 | 0.42–1.72 | 0.66 | 0.84 | 0.26–2.74 | 0.78 | 1.00 | 0.50–2.00 | 1.00 |
| Tri-fascicular block | 1.12 | 0.39–3.19 | 0.83 | 0.68 | 0.08–5.52 | 0.72 | 1.15 | 0.40–3.28 | 0.79 |
| Non-elective procedure | 0.83 | 0.17–3.97 | 0.82 | 3.53 | 0.67–18.63 | 0.14 | 1.48 | 0.38–5.78 | 0.58 |
| Non-femoral approach | 1.63 | 0.55–4.86 | 0.38 | 1.02 | 0.12–8.48 | 0.98 | 1.68 | 0.56–4.99 | 0.35 |
| General anesthesia (vs. conscious sedation) | 1.84 | 0.88–3.86 | 0.10 | 2.82 | 0.90–8.80 | 0.074 | 2.19 | 1.06–4.55 | **0.035** |
| Valve size: ≥29 mm (vs. ≤23 mm) | 0.60 | 0.28–1.29 | 0.19 | 7.12 | 0.88–57.37 | 0.065 | 1.11 | 0.51–2.41 | 0.79 |
| Valve size: 26 mm (vs. ≤23 mm) | 0.99 | 0.47–2.09 | 0.97 | 4.73 | 0.53–41.83 | 0.16 | 1.29 | 0.58–2.88 | 0.53 |
| Pre-dilation | 1.89 | 0.96–3.72 | 0.064 | 6.18 | 2.14–17.85 | **0.001** | 3.13 | 1.61–6.08 | **0.001** |
| Post-dilation | 1.17 | 0.63–2.16 | 0.62 | 0.43 | 0.13–1.37 | 0.15 | 1.00 | 0.54–1.86 | 1.00 |
| Oversizing, %, per 1% increase | 1.00 | 0.98–1.02 | 1.00 | 1.00 | 0.98–1.03 | 0.88 | 1.00 | 0.98–1.02 | 0.91 |
| Implantation depth relative to NCC, per 1mm increase | 1.28 | 1.09–1.50 | **0.003** | 1.56 | 1.17–2.09 | **0.002** | 1.40 | 1.19–1.66 | **<0.001** |

Abbreviations as in Supplemental Tables 2-4.

**Supplemental Table 7. Predictors of HAVB/CHB and 30-day PPM/ICD requirement post-TAVI with a balloon-expandable valve in patients with pre-existing RBBB**

|  | **Univariable analyses** | | | **Multivariable analyses** | | |
| --- | --- | --- | --- | --- | --- | --- |
|  | **OR** | **95% CI** | **P value** | **OR** | **95% CI** | **P value** |
| **Procedural HAVB/CHB (n=248)** |  |  |  |  |  |  |
| STS risk score, per 1% increase | 1.09 | 1.02–1.16 | 0.012 | 1.08 | 1.00–1.15 | 0.036 |
| Aortic valve area, per 0.1 cm^2^ decrease | 1.24 | 1.03–1.48 | 0.022 | 1.21 | 1.00–1.46 | 0.045 |
| Implantation depth relative to NCC, per 1 mm increase | 1.28 | 1.09–1.50 | 0.003 | 1.26 | 1.07–1.49 | 0.006 |
| **Delayed HAVB/CHB (n=196)*** |  |  |  |  |  |  |
| Pre-dilation | 6.18 | 2.14–17.85 | 0.001 | 4.06 | 1.31–12.61 | 0.015 |
| Implantation depth relative to NCC, per 1 mm increase | 1.56 | 1.17–2.09 | 0.002 | 1.39 | 1.03–1.89 | 0.034 |
| **30-day PPM/ICD requirement (n=248)** |  |  |  |  |  |  |
| LVOT calcification | 2.12 | 1.09–4.09 | 0.026 | 1.75 | 0.87–3.52 | 0.12 |
| General anesthesia (vs. conscious sedation) | 2.19 | 1.06–4.55 | 0.035 | 0.77 | 0.31–1.90 | 0.57 |
| Pre-dilation | 3.13 | 1.61–6.08 | 0.001 | 2.22 | 1.03–4.80 | 0.042 |
| Implantation depth relative to NCC, per 1 mm increase | 1.40 | 1.19–1.66 | <0.001 | 1.35 | 1.12–1.62 | 0.001 |

Predictors were examined in multivariable logistic regression models including variables with p value at <0.05 in univariable models (**Supplemental Table 5**). In multivariable models, missing data for aortic valve area, implantation depth, and LVOT calcification were handled with multiple imputation. *Includes patients who did not develop procedural HAVB/CHB. Abbreviations as in Supplemental Tables 2-4.

**Supplemental Table 8. Predictive values of pre-dilation and implantation depth for HAVB/CHB and 30-day PPM/ICD**

|  | **No. of events (%)** | **Sensitivity** | **Specificity** | **PPV** | **NPV** |
| --- | --- | --- | --- | --- | --- |
| **Procedural HAVB/CHB (n=259)** |  |  |  |  |  |
| Pre-dilation | 18/61 (29.5%) | 32.7% | 78.9% | 29.5% | 81.3% |
| Implantation depth >1.0 mm | 45/191 (23.6%) | 81.8% | 28.4% | 23.6% | 85.3% |
| Implantation depth >2.0 mm | 39/137 (28.5%) | 70.9% | 52.0% | 28.5% | 86.9% |
| Implantation depth >3.0 mm | 29/97 (29.9%) | 52.7% | 66.7% | 29.9% | 84.0% |
| **Delayed HAVB/CHB (n=204)** |  |  |  |  |  |
| Pre-dilation | 10/43 (23.3%) | 58.8% | 82.4% | 23.3% | 95.7% |
| Implantation depth >1.0 mm | 15/146 (10.3%) | 88.2% | 29.9% | 10.3% | 96.6% |
| Implantation depth >2.0 mm | 14/98 (14.3%) | 82.4% | 55.1% | 14.3% | 97.2% |
| Implantation depth >3.0 mm | 11/68 (16.2%) | 64.7% | 69.5% | 16.2% | 95.6% |
| **30-day PPM/ICD requirement (n=259)** |  |  |  |  |  |
| Pre-dilation | 24/61 (37.7%) | 41.1% | 81.3% | 37.7% | 83.3% |
| Implantation depth >1.0 mm | 49/191 (25.7%) | 87.5% | 30.0% | 25.7% | 89.7% |
| Implantation depth >2.0 mm | 42/137 (30.7%) | 75.0% | 53.2% | 30.7% | 88.5% |
| Implantation depth >3.0 mm | 33/97 (34.0%) | 58.9% | 68.5% | 34.0% | 85.8% |

PPV = positive predictive value; NPV = negative predictive value; other abbreviations as in Supplemental Tables 2 and 3.
